# Supplementary material for: Trauma-affected refugees treated with basic body awareness therapy or mixed physical activity as augmentation to treatment as usual—A pragmatic randomised controlled trial
Source: PLoS One. 2020 Mar 12;15(3):e0230300. doi: 10.1371/journal.pone.0230300 (PMC7067472; doi:10.1371/journal.pone.0230300)
Supplement: S1 Table — (DOCX) [file pone.0230300.s001.docx]

***S1 Table:* Score differences between pre-treatment ratings and post-treatment ratings in the intention-to-treat population**

| Rating | Groups and differences | Mean pre-treatment score (SE) | Mean post-treatment score (SE) | Difference (SE) | P-value |
| --- | --- | --- | --- | --- | --- |
| SCL-90 | Control | 2.50 (0.08) | 2.36 (0.10) | **-0.14 (0.09)** | 0.1139 |
|  | BBAT | 2.55 (0.07) | 2.46 (0.10) | **-0.09 (0.09)** | 0.2942 |
|  | Mixed physical activity | 2.50 (0.08) | 2.47 (0.11) | **-0.03 (0.10)** | 0.7369 |
|  | Difference, p-value | 0.8699 | 0.7232 | 0.7337 | - |
| VAS | Control | 6.67 (0.22) | 6.67 (0.27) | *0.00 (0.23)* | 0.9697 |
|  | BBAT | 6.86 (0.22) | 6.49 (0.26) | **0.37 (0.20)** | 0.0684 |
|  | Mixed physical activity | 6.69 (0.25) | 6.41 (0.30) | **0.28 (0.28)** | 0.3154 |
|  | Difference, p-value | 0.8173 | 0.7959 | 0.4861 | - |
| HoNOS | Control | 1.09 (0.04) | 0.91 (0.05) | **-0.18 (0.05)** | 0.0009** |
|  | BBAT | 1.10 (0.04) | 0.96 (0.05) | **-0.14 (0.06)** | 0.0081** |
|  | Mixed physical activity | 1.14 (0.04) | 0.90 (0.06) | **-0.24 (0.06)** | 0.0001** |
|  | Difference, p-value | 0.6956 | 0.7102 | 0.5366 | - |
| BPI shaded | Control | 27.44 (1.75) | 22.12 (1.89) | **-5.32 (2.00)** | 0.0078** |
|  | BBAT | 31.30 (2.06) | 25.34 (2.31) | **-5.95 (2.19)** | 0.0065** |
|  | Mixed physical activity | 28.08 (1.99) | 24.09 (2.37) | **-3.99 (2.10)** | 0.0570 |
|  | Difference, p-value | 0.3342 | 0.5427 | 0.8021 | - |
| MAIA noticing | Control | 3.78 (0.09) | 3.73 (0.09) | *-0.05 (0.11)* | 0.6634 |
|  | BBAT | 3.56 (0.09) | 3.52 (0.10) | *-0.04 (0.11)* | 0.7411 |
|  | Mixed physical activity | 3.53 (0.10) | 3.52 (0.09) | *-0.01 (0.11)* | 0.8873 |
|  | Difference, p-value | 0.1371 | 0.1814 | 0.9764 | - |
| MAIA not-distracting | Control | 2.43 (0.14) | 2.69 (0.14) | **0.26 (0.22)** | 0.2404 |
|  | BBAT | 2.19 (0.13) | 2.55 (0.13) | **0.36 (0.22)** | 0.0981 |
|  | Mixed physical activity | 2.16 (0.13) | 2.72 (0.15) | **0.56 (0.22)** | 0.0107* |
|  | Difference, p-value | 0.2922 | 0.6566 | 0.6145 | - |
| MAIA not-worrying | Control | 1.20 (0.10) | 3.50 (0.08) | **2.30 (0.13)** | 0.0000** |
|  | BBAT | 1.39 (0.10) | 3.38 (0.10) | **1.99 (0.15)** | 0.0000** |
|  | Mixed physical activity | 1.17 (0.10) | 3.42 (0.09) | **2.25 (0.15)** | 0.0000** |
|  | Difference, p-value | 0.1995 | 0.6188 | 0.2397 | - |
| MAIA attention regulation | Control | 2.45 (0.11) | 2.50 (0.14) | **0.05 (0.14)** | 0.6803 |
|  | BBAT | 2.46 (0.11) | 2.37 (0.13) | *-0.09 (0.13)* | 0.4786 |
|  | Mixed physical activity | 2.58 (0.13) | 2.22 (0.14) | *-0.36 (0.15)* | 0.0155* |
|  | Difference, p-value | 0.7133 | 0.3489 | 0.1206 | - |
| MAIA emotional awareness | Control | 3.50 (0.12) | 3.67 (0.11) | **0.17 (0.12)** | 0.1796 |
|  | BBAT | 3.47 (0.10) | 3.45 (0.10) | *-0.02 (0.11)* | 0.8719 |
|  | Mixed physical activity | 3.49 (0.11) | 3.44 (0.14) | *-0.05 (0.14)* | 0.7181 |
|  | Difference, p-value | 0.9783 | 0.3016 | 0.4262 | - |
| MAIA self-regulation | Control | 1.92 (0.13) | 2.02 (0.14) | **0.10 (0.13)** | 0.4476 |
|  | BBAT | 1.80 (0.11) | 2.11 (0.13) | **0.31 (0.12)** | 0.0079** |
|  | Mixed physical activity | 1.81 (0.13) | 2.14 (0.15) | **0.33 (0.13)** | 0.0107* |
|  | Difference, p-value | 0.7586 | 0.8217 | 0.3451 | - |
| MAIA body listening | Control | 2.28 (0.15) | 2.45 (0.17) | **0.17 (0.18)** | 0.3249 |
|  | BBAT | 2.32 (0.14) | 2.27 (0.14) | *-0.05 (0.14)* | 0.7288 |
|  | Mixed physical activity | 2.43 (0.16) | 2.40 (0.17) | *-0.03 (0.17)* | 0.8914 |
|  | Difference, p-value | 0.7763 | 0.6782 | 0.5873 | - |
| MAIA trusting | Control | 2.50 (0.16) | 2.35 (0.16) | *-0.14 (0.17)* | 0.3872 |
|  | BBAT | 2.44 (0.14) | 2.35 (0.15) | *-0.09 (0.18)* | 0.6406 |
|  | Mixed physical activity | 2.26 (0.15) | 2.36 (0.19) | *-0.10 (0.19)* | 0.6052 |
|  | Difference, p-value | 0.5093 | 1.0000 | 0.6169 | - |
| DEMMI | Control | 76.51 (2.26) | 69.93 (2.74) | *-6.58 (2.47)* | 0.0077** |
|  | BBAT | 81.49 (3.01) | 74.75 (4.40) | *-6.74 (4.65)* | 0.1469 |
|  | Mixed physical activity | 76.28 (2.04) | 75.77 (2.22) | *-0.51 (1.67)* | 0.7626 |
|  | Difference, p-value | 0.3164 | 0.2434 | 0.0837 | - |
| SFT 1: chair stand  (number of repetitions) | Control | 7.64 (0.53) | 7.38 (0.60) | *-0.26 (0.48)* | 0.5865 |
|  | BBAT | 7.65 (0.44) | 7.68 (0.53) | **0.03 (0.35)** | 0.9321 |
|  | Mixed physical activity | 7.45 (0.43) | 7.35 (0.48) | *-0.10 (0.27)* | 0.7193 |
|  | Difference, p-value | 0.9397 | 0.8890 | 0.8841 |  |
| SFT 2: arm curl test  (number of repetitions) | Control | 10.61 (0.67) | 10.92 (0.78) | **0.30 (0.53)** | 0.5583 |
|  | BBAT | 10.50 (0.57) | 10.89 (0.67) | **0.39 (0.41)** | 0.3546 |
|  | Mixed physical activity | 9.78 (0.57) | 9.69 (0.65) | *-0.09 (0.43)* | 0.8374 |
|  | Difference, p-value | 0.5568 | 0.3408 | 0.7115 |  |
| SFT 3: 2-minute step test  (number of repetitions) | Control | 37.04 (3.01) | 39.52 (4.51) | **2.48 (3.86)** | 0.5214 |
|  | BBAT | 35.18 (2.55) | 37.40 (2.91) | **2.22 (1.93)** | 0.2493 |
|  | Mixed physical activity | 35.49 (2.50) | 37.08 (4.22) | **1.59 (2.91)** | 0.5862 |
|  | Difference, p-value | 0.8852 | 0.9080 | 0.9778 |  |
| SFT 4: chair sit-and-reach-test  (cm +/-) | Control | -5.80 (1.98) | -5.28 (2.55) | **0.52 (3.29)** | 0.8775 |
|  | BBAT | -5.10 (1.97) | -8.35 (2.16) | *-3.25 (1.68)* | 0.0538 |
|  | Mixed physical activity | -7.10 (1.89) | -4.50 (2.55) | **2.60 (2.98)** | 0.3830 |
|  | Difference, p-value | 0.7559 | 0.4589 | 0.1883 |  |
| SFT 5: back scratch test  (cm +/-) | Control | -6.25 (2.29) | -1.95 (2.83) | **4.29 (3.68)** | 0.2433 |
|  | BBAT | -4.53 (1.74) | -5.12 (2.16) | *-0.59 (1.38)* | 0.6719 |
|  | Mixed physical activity | -6.18 (1.81) | -3.39 (2.73) | **2.79 (2.73)** | 0.3076 |
|  | Difference, p-value | 0.7590 | 0.6631 | 0.3038 |  |
| SFT 6: 2.45m up-and-go test  (sec) | Control | 8.92 (0.50) | 9.67 (0.58) | *0.75 (0.56)* | 0.1784 |
|  | BBAT | 8.28 (0.62) | 8.89 (0.78) | *0.61 (0.48)* | 0.2014 |
|  | Mixed physical activity | 9.91 (0.67) | 10.05 (0.76) | *0.14 (0.54)* | 0.7922 |
|  | Difference, p-value | 0.1998 | 0.5560 | 0.7041 |  |
| DGI | Control | 19.81 (0.50) | 19.27 (0.66) | *-0.54 (0.57)* | 0.3475 |
|  | BBAT | 20.96 (0.41) | 20.98 (0.50) | **0.02 (0.34)** | 0.9539 |
|  | Mixed physical activity | 20.24 (0.46) | 20.41 (0.57) | **0.17 (0.46)** | 0.7109 |
|  | Difference, p-value | 0.1834 | 0.1197 | 0.6075 |  |

SE=standard error

***** p ≤ 0.05

** p ≤0.01

**Bold =** Improvement, *Italic* = Deterioration

SCL-90 = 1-4 (1 best score), VAS = 0–10 (0 best score), HoNOS = 0-4 (0 best score), BPI shaded = 0-100 (0 best score), MAIA = 0-5 (5 best score), DEMMI = 0-100 (100 best score), SFT-1-6 = each subscale with a normal range of score defined according to gender and age; SFT-1-5: the higher the better score; SFT-6: the lower the better score. DGI = 0-24 (24 best score).

*SCL-90* Symptom Chechlist-90, *VAS* Visual Analogue Scale, *HoNOS* Health of Nation Outcome Scales, *BPI* Brief Pain Inventory, *MAIA* Multidimensional Assessment of Interoceptive Awareness, *DEMMI* de Morton Mobility Index, *SFT 1-6* Senior Fitness Test (subscales 1-6), *DGI* Dynamic Gait Index.

Outline of pre-and post-treatment rating scores for the intention-to-treat population. The p-values refer to the significance of differences between groups at pre-treatment and at post-treatment, between pre- and post- treatment ratings in each group and the significance of group differences in the difference between pre-and post- treatment ratings (corresponding to the interaction between intervention group and rating time).
