# Supplementary material for: Trauma-affected refugees treated with basic body awareness therapy or mixed physical activity as augmentation to treatment as usual—A pragmatic randomised controlled trial
Source: PLoS One. 2020 Mar 12;15(3):e0230300. doi: 10.1371/journal.pone.0230300 (PMC7067472; doi:10.1371/journal.pone.0230300)
Supplement: S2 Table — (DOCX) [file pone.0230300.s002.docx]

**S2 Table** **Score differences between pre-treatment ratings and post-treatment ratings in the per-protocol population**

| Rating | Groups and differences | Mean pre-treatment score (SE) | Mean post-treatment score (SE) | Difference (SE) | P-value |
| --- | --- | --- | --- | --- | --- |
| HTQ | Control | 3.17 (0.04) | 2.96 (0.06) | **-0.21 (0.06)** | 0.0003** |
|  | Basic body awareness therapy | 3.22 (0.05) | 3.02 (0.08) | **-0.21 (0.06)** | 0.0006** |
|  | Mixed physical activity | 3.21(0.06) | 3.02 (0.08) | **-0.19 (0.07)** | 0.0100** |
|  | Difference, p-value | 0.6884 | 0.769 | 0.9769 |  |
| HSCL-25 | Control | 3.02 (0.05) | 2.80 (0.07) | **-0.22 (0.07)** | 0.0016** |
|  | Basic body awareness therapy | 3.01 (0.07) | 2.88 (0.09) | **-0.13 (0.07)** | 0.0639 |
|  | Mixed physical activity | 2.97 (0.07) | 2.90 (0.09) | **-0.70 (0.09)** | 0.4049 |
|  | Difference, p-value | 0.8588 | 0.6441 | 0.4142 |  |
| SCL-90 | Control | 2.50(0.08) | 2.36 (0.10) | **-0.14 (0.09)** | 0.1050 |
|  | Basic body awareness therapy | 2.60 (0.09) | 2.43 (0.12) | **-0.17 (0.10)** | 0.0847 |
|  | Mixed physical activity | 2.62 (0.11) | 2.50 (0.14) | **-0.13 (0.11)** | 0.2382 |
|  | Difference, p-value | 0.5427 | 0.7017 | 0.9456 |  |
| SDS | Control | 22.62 (0.62) | 22.36 (0.84) | **-0.26 (0.85)** | 0.7593 |
|  | Basic body awareness therapy | 22.19 (0.84) | 21.87 (1.07) | **-0.32 (0.89)** | 0.7150 |
|  | Mixed physical activity | 24.26 (0.65) | 21.93 (0.98) | **-2.33 (0.92)** | 0.0118* |
|  | Difference, p-value | 0.0830 | 0.9189 | 0.1863 |  |
| WHO | Control | 14.94 (1.59) | 23.27 (2.56) | **8.33 (2.36)** | 0.0004** |
|  | Basic body awareness therapy | 15.78 (2.03) | 23.04 (3.05) | **7.26 (2.45)** | 0.0030* |
|  | Mixed physical activity | 14.90 (1.95) | 24.69 (3.11) | **9.79 (3.07)** | 0.0014** |
|  | Difference, p-value | 0.9376 | 0.9176 | 0.8134 |  |
| Ham-D | Control | 22.73 (0.65) | 21·75 (0.77) | **-0.98 (0.77)** | 0.1976 |
|  | Basic body awareness therapy | 22.67 (0.75) | 21.65 (1.07) | **-1.02(0.85)** | 0.2289 |
|  | Mixed physical activity | 22.34 (0.90) | 21.41 (0.93) | **-0.93 (0.86)** | 0.2788 |
|  | Difference, p-value | 0.9374 | 0.9614 | 0.9974 |  |
| Ham-A | Control | 26.82 (0.85) | 26.79 (0.96) | **-0.03 (1.06)** | 0.9744 |
|  | Basic body awareness therapy | 26.76 (0.92) | 25.44 (1.24) | **-1.32 (1.10)** | 0.2319 |
|  | Mixed physical activity | 27.09 (1.23) | 27.61 (1.27) | *0.52 (1.08)* | 0.6318 |
|  | Difference, p-value | 0.9757 | 0.4602 | 0.4760 |  |
| VAS | Control | 6.67 (0.22) | 6.67(0.27) | *0.01 (0.23)* | 0.9813 |
|  | Basic body awareness therapy | 6.84 (0.28) | 6.57(0.34) | **-0.27 (0.25)** | 0.2629 |
|  | Mixed physical activity | 6.98(0.30) | 6.71(0.35) | **-0.27 (0.35)** | 0.4433 |
|  | Difference, p-value | 0.7052 | 0.9560 | 0.6809 |  |
| GAF-F | Control | 51.63 (0.87) | 55.05 (1.22) | **3.42 (1.31)** | 0.0087 |
|  | Basic body awareness therapy | 51.16 (1.01) | 53.60 (1.26) | **2.43 (1.16)** | 0.0355* |
|  | Mixed physical activity | 51.12 (0.99) | 54.58 (1.67) | **3.46 (1.43)** | 0.0157** |
|  | Difference, p-value | 0.9111 | 0.7048 | 0.8019 |  |
| GAF-S | Control | 51.27 (0.68) | 55.88 (1.16) | **4.62 (1.20)** | 0.0001** |
|  | Basic body awareness therapy | 50.54 (0.81) | 53.41 (1.07) | **2.87 (0.97)** | 0.0031** |
|  | Mixed physical activity | 50.63 (0.82) | 54.58 (1.66) | **3.95 (1.53)** | 0.0096** |
|  | Difference, p-value | 0.7433 | 0.2915 | 0.5142 |  |
| HoNOS | Control | 1.09 (0.04) | 0.91 (0.05) | **-0.18 (0.05)** | 0.0009** |
|  | Basic body awareness therapy | 1.15 (0.05) | 0.93 (0.06) | **-0.21 (0.07)** | 0.0021** |
|  | Mixed physical activity | 1.09 (0.06) | 0.90 (0.07) | **-0.18 (0.08)** | 0.0197** |
|  | Difference, p-value | 0.6537 | 0.9398 | 0.9153 |  |
| BPI shaded | Control | 27.44 (1.75) | 22.12 (1.89) | **-5.32 (2.00)** | 0.0078** |
|  | Basic body awareness therapy | 34.81 (2.75) | 31.69 (3.03) | **-3.12 (2.74)** | 0.2562 |
|  | Mixed physical activity | 31.00 (2.48) | 34.17 (2.90) | *3.17 (2.02)* | 0.1156 |
|  | Difference, p-value | 0.0693 | 0.0005 | 0.0093** |  |
| BPI severity | Control | 6.37 (0.18) | 6.58 (0.21) | *0.21 (0.19)* | 0.2836 |
|  | Basic body awareness therapy | 5.98 (0.29) | 6.22 (0.28) | *0.24 (0.22)* | 0.2773 |
|  | Mixed physical activity | 6.56 (0.26) | 6.87 (0.28) | *0.31 (0.22)* | 0.1565 |
|  | Difference, p-value | 0.3273 | 0.2787 | 0.9438 |  |
| BPI interference | Control | 7.82 (0.17) | 7.54 (0.21) | **-0.27 (0.23)** | 0.2302 |
|  | Basic body awareness therapy | 6.95 (0.32) | 6.62 (0.36) | **-0.33 (0.30)** | 0.2579 |
|  | Mixed physical activity | 7.41 (0.31) | 7.28 (0.32) | **-0.13 (0.27)** | 0.6176 |
|  | Difference, p-value | 0.0481* | 0.0886 | 0.8682 |  |
| MAIA noticing | Control | 3.78 (0.09) | 3.73 (0.09) | *-0.05 (0.11)* | 0.6567 |
|  | Basic body awareness therapy | 3.65 (0.12) | 3.48 (0.12) | *-0.17 (0.13)* | 0.1893 |
|  | Mixed physical activity | 3.48 (0.10) | 3.53 (0.09) | **0.05 (0.12)** | 0.6562 |
|  | Difference, p-value | 0.1035 | 0.1724 | 0.4464 |  |
| MAIA not-distracting | Control | 2.43 (0.14) | 2.69 (0.14) | **0.24 (0.22)** | 0.2407 |
|  | Basic body awareness therapy | 2.22 (0.17) | 2.57 (0.15) | **0.35 (0.28)** | 0.2037 |
|  | Mixed physical activity | 2.13 (0.15) | 2.78 (0.17) | **0.65 (0.26)** | 0.0135* |
|  | Difference, p-value | 0.3178 | 0.6522 | 0.5113 |  |
| MAIA not-worrying | Control | 1.20 (0.10) | 3.50 (0.08) | **2.30 (0.13)** | 0.0000** |
|  | Basic body awareness therapy | 1.30 (0.12) | 3.41 (0.12) | **2.11 (0.18)** | 0.0000** |
|  | Mixed physical activity | 1.11 (0.12) | 3.42 (0.10) | **2.31 (0.18)** | 0.0000** |
|  | Difference, p-value | 0.5259 | 0.7266 | 0.6460 |  |
| MAIA attention regulation | Control | 2.45 (0.11) | 2.50 (0.14) | 0.05 (0.14) | 0.6880 |
|  | Basic body awareness therapy | 2.49 (0.15) | 2.34 (0.16) | *-0.15 (0.17)* | 0.3882 |
|  | Mixed physical activity | 2.50 (0.15) | 2.18 (0.15) | *- 0.32 (0.17)* | 0.0582 |
|  | Difference, p-value | 0.9550 | 0.2953 | 0.2273 |  |
| MAIA emotional awareness | Control | 3.50 (0.12) | 3.67 (0.11) | **0.17 (0.12)** | 0.1787 |
|  | Basic body awareness therapy | 3.44 (0.13) | 3.46 (0.12) | **0.02 (0.12)** | 0.9161 |
|  | Mixed physical activity | 3.35 (0.13) | 3.42 (0.15) | **0.07 (0.15)** | 0.6456 |
|  | Difference, p-value | 0.6817 | 0.3054 | 0.6843 |  |
| MAIA self- regulation | Control | 1.92 (0.13) | 2.02 (0.14) | **0.10 (0.13)** | 0.4490 |
|  | Basic body awareness therapy | 1.83 (0.15) | 2.09 (0.15) | **0.26 (0.14)** | 0.0657 |
|  | Mixed physical activity | 1.73 (0.15) | 2.05 (0.16) | **0.32 (0.14)** | 0.0232* |
|  | Difference, p-value | 0.6422 | 0.9336 | 0.4566 |  |
| MAIA body listening | Control | 2.28 (0.15) | 2.45 (0.17) | **0.17 (0.18)** | 0.3262 |
|  | Basic body awareness therapy | 2.26 (0.18) | 2.24 (0.17) | *-0.02 (0.17)* | 0.9158 |
|  | Mixed physical activity | 2.42 (0.19) | 2.34 (0.19) | *-0.08 (0.19)* | 0.6858 |
|  | Difference, p-value | 0.8025 | 0.6873 | 0.5908 |  |
| MAIA trusting | Control | 2.50 (0.16) | 2.36 (0.16) | *-0.14 (0.17)* | 0.4209 |
|  | Basic body awareness therapy | 2.44 (0.18) | 2.36 (0.18) | *-0.08 (0.20)* | 0.6725 |
|  | Mixed physical activity | 2.31 (0.19) | 2.14 (0.21) | *-0.17 (0.20)* | 0.4019 |
|  | Difference, p-value | 0.7508 | 0.6732 | 0.9592 |  |
| DEMMI | Control | 76.51 (2.26) | 69.93 (2.74) | *-6.58 (2.47)* | 0.0077** |
|  | Basic body awareness therapy | 82.45 (3.12) | 75.28 (4.56) | *-7.17 (3.98)* | 0.0713 |
|  | Mixed physical activity | 73.91 (2.52) | 74.20 (2.56) | **0.29 (1.85)** | 0.8756 |
|  | Difference, p-value | 0.1001 | 0.4313 | 0.0419* |  |
| SFT 1: chair stand  (number of repetitions) | Control | 7.64 (0.53) | 7.38 (0.60) | -*0.26* (0.49) | 0.5884 |
|  | Basic body awareness therapy | 7.48 (0.52) | 7.73 (0.61) | *-0.25* (0.39) | 0.5198 |
|  | Mixed physical activity | 7.00 (0.59) | 6.87 (0.63) | *-0.13* (0.32) | 0.7030 |
|  | Difference, p-value | 0.7045 | 0.6251 | 0.6595 |  |
| SFT 2: arm curl test  (number of repetitions) | Control | 10.61 (0.67) | 10.91 (0.78) | **0.30 (0.53)** | 0.5671 |
|  | Basic body awareness therapy | 10.06 (0.66) | 10.54 (0.81) | **0.48 (0.50)** | 0.3470 |
|  | Mixed physical activity | 9.09 (0.68) | 9.05 (0.75) | *-0.04 (0.51)* | 0.9379 |
|  | Difference, p-value | 0.2754 | 0.1890 | 0.7666 |  |
| SFT 3: 2-minute step test  (number of repetitions) | Control | 37.05 (3.·02) | 39.49 (4.51) | **2.44 (3.86)** | 0.5264 |
|  | Basic body awareness therapy | 34.99 (3·.06) | 38.20 (3.59) | **3.21 (2.20)** | 0.1450 |
|  | Mixed physical activity | 32.68 (3.21) | 33.97 (4.70) | **1.29 (2.81)** | 0.6476 |
|  | Difference, p-value | 0.6122 | 0.6711 | 0.8648 |  |
| SFT 4: chair sit-and-reach-test  (cm +/-) | Control | -5.80 (1.98) | -5.28 (2.55) | **0.52 (3.29)** | 0.8748 |
|  | Basic body awareness therapy | -6.49 (2.42) | -8.89 (2.49) | *-2.40 (1.67)* | 0.1508 |
|  | Mixed physical activity | -7.95 (2.48) | -7.78 (3.03) | **0.18 (3.64)** | 0.9616 |
|  | Difference, p-value | 0.7934 | 0.5872 | 0.6440 |  |
| SFT 5: back scratch test  (cm +/-) | Control | -6.24 (2.29) | -1.95 (2.83) | **4.29 (3.68)** | 0.2432 |
|  | Basic body awareness therapy | -5.02 (2.20) | -6.30 (2.58) | -1.28 (1.54) | 0.4084 |
|  | Mixed physical activity | -6.14 (2.17) | -6.79 (3.25) | -0.65 (3.03) | 0.8294 |
|  | Difference, p-value | 0.9112 | 0.4234 | 0.3768 |  |
| SFT 6: 2.45m up-and-go test  (sec) | Control | 8.92 (0.50) | 9.67 (0.58) | *0.75 (0.56)* | 0.1781 |
|  | Basic body awareness therapy | 8.46 (0.83) | 9.07 (0.97) | *0.61 (0.57)* | 0.2837 |
|  | Mixed physical activity | 10.49 (0.85) | 10.22 (0.87) | **- 0.27 (0.59)** | 0.6507 |
|  | Difference, p-value | 0.1821 | 0.6728 | 0.4056 |  |
| DGI | Control | 19.81 (0.50) | 19.27 (0.66) | *-0.54 (0.57)* | 0.3470 |
|  | Basic body awareness therapy | 20.83 (0.47) | 21.15 (0.57) | **0.32 (0.37)** | 0.3764 |
|  | Mixed physical activity | 19.69 (0.65) | 20.35 (0.65) | **0.66 (0.52)** | 0.2025 |
|  | Difference, p-value | 0.2221 | 0.0999 | 0.2788 |  |

SE=standard error

***** p ≤ 0.05

** p ≤0.01

**Bold =** Improvement, *Italic* = Deterioration

HTQ, HSCL-25, SCL = 1–4 (1 best score), SCL-90=1-4 (1 best score), SDS = 0–10 (0 best score), WHO-5 = 0–100 (100 best score), HAM-D = 0–52 (0 best score), HAM-A = 0–56 (0 best score), VAS = 0–10 (0 best score), GAF-S/-F = 0-100 (100 best score), HoNOS = 0-4 (0 best score), BPI shaded = 0-100 (0 best score), BPI severity/interference = 0-10 (0 best score), MAIA = 0-5 (5 best score), DEMMI = 0-100 (100 best score), SFT-1-6 = each subscale with a normal range of score defined according to gender and age; SFT-1-5: the higher the better score; SFT-6: the lower the better score. DGI= 0-24 (24 best score).

*HTQ* Harvard Trauma Questionnaire, *HSCL-25* Hopkins Symptom Checklist-25, *SCL-90* Symptom Chechlist-90, *SDS* Sheehan Disability Scale, *WHO-5* WHO-5 Well Being Index, *HAM-D/-A* Hamilton Depression/Anxiety Rating scales, *VAS* Visual Analogue Scale, *GAF-F/-S* Global assessment of Functioning (Symptom/Function), *HoNOS* Health of Nation Outcome Scales, *BPI severity/interference* Brief Pain Inventory severity/interference, *MAIA* Multidimensional Assessment of Interoceptive Awareness, *DEMMI* de Morton Mobility Index, *SFT 1-6* Senior Fitness Test (subscales 1-6), *DGI* Dynamic Gait Index.

Outline of pre-and post-treatment rating scores for the per-protocol population. The p-values refer to the significance of differences between groups at pre-treatment and at post-treatment, between pre- and post- treatment ratings in each group and the significance of group differences in the difference between pre-and post- treatment ratings (corresponding to the interaction between intervention group and rating time).
