# Supplementary material for: Trauma-affected refugees treated with basic body awareness therapy or mixed physical activity as augmentation to treatment as usual—A pragmatic randomised controlled trial
Source: PLoS One. 2020 Mar 12;15(3):e0230300. doi: 10.1371/journal.pone.0230300 (PMC7067472; doi:10.1371/journal.pone.0230300)
Supplement: S1 Protocol — (DOC) [file pone.0230300.s004.doc]

Research Protocol:

**Treatment of traumatised refugees: the effect of Basic Body Awareness Therapy versus mixed physical activity as add-on treatment.**

**A Randomised Controlled Trial.**

Protocol code: PTF4

Sponsor: Jessica Carlsson Lohmann

Investigator: Maja Sticker Nordbrandt

Competence Centre for Transcultural Psychiatry,

Psychiatric Center Ballerup, Gentofte,

Niels Andersens Vej 65, Opgang 32 Stuen, 2900 Hellerup, Denmark.

**TABLE OF CONTENTS**

**1.1 Background of the trial**

**1.2 Traumatised refugees**

**1.3 About the clinic**

**1.4 Previous research**

**1.5 The scientific importance of the present study**

**1.6 Theoretical framework of the study**

**1.6.1 Physical activity and the physiology**

**1.6.2 Physical activity and the autonomous nervous system**

**1.6.3 Basic Body Awareness Therapy**

**1.6.4 Study of the feasibility of BBAT for traumatised refugees**

**1.6.5 Mixed physical activity as intervention for traumatised**

**1.1.4 Theoretical framework of the study**

**1.7 The present study**

**1.8 Aims of the study**

**1.9 Hypotheses**

**2. Research methods**

**2.1 Trial design**

**2.2 Inclusion criteria**

**2.3 Exclusion criteria**

**2.4 Outcome measures**

**2.5 Randomisation**

**2.6 Blinding**

**2.7 Drop-out**

**2.8 Extraction criteria**

**2.9 Programme fidelity**

**2.10 Representativity**

**3. Course of treatment**

**3.1 Referral**

**3.2 Referral interview**

**3.3 First psychological assessment**

**3.4 Physical activity**

**3.5 Medical treatment**

**3.6 Psychologist consultations**

**3.7 Social counsellor**

**3.8 Evaluation and follow-up**

**3.9 The patient group**

**3.10 Interpretation**

**3.11 Variability in the treatment programme**

**3.12 Treatment responsibility**

**3.13 Collaboration with the primary sector**

**4. The physical activities**

**4.1 Basic Body Awareness Therapy (BBAT)**

**4.2 Mixed physical activity (MPA)**

**4.3 Home exercises**

**4.4 Adverse events and side effects**

**5. Data collection**

**5.1 Rating**

**5.2 Frequency of rating**

**5.3 Risk factors**

**5.4 Satisfaction study**

**5.5 Drop-out analysis**

**6. Safety, quality assurance and responsibility**

**6.1 Responsible for data collection**

**6.2 Data security**

**6.3 Source data**

**6.4 Quality assurance**

**6.5 Treatment responsibility after the trial**

**7. Data analysis**

**7.1 Size of material**

**7.2 Drop-out analysis**

**7.3 Power calculations**

**7.4 Data processing**

**8. Ethical considerations**

**8.1 Informed consent**

**8.2 Ethical aspects in randomisation**

**8.3 Right of access to patient records**

**8.4 Disadvantages of the trial**

**8.5 Insurance and compensation**

**9. Project organisation**

**9.1 Project initiation**

**9.2 Collaboration partners**

**9.3 Time schedule**

**9.4 Publications**

**9.5 Financing**

**9.6 Reports**

**10. References**

**Annex 1**

**Annex 2**

**List of abbreviations**

BBAT Basic Body Awareness therapy

ACC Anterior cingulate cortex

ACT Acceptance and Commitment Therapy

CRF Case Report Form

CTP Competence Centre for Transcultural Psychiatry

GAF Global Assessment of Functioning

HAM- A Hamilton Anxiety Rating Scale

HAM-D Hamilton Depression Rating Scale

HRV Heart Rate Variability

HSCL-25 Hopkin’s Symptom Check List

HTQ Harvard Trauma Questionnaire

ICD-10 International Classification of Diseases-10

Manual-based CBT

Manual-based Cognitive Behavioural Therapy

MAIA Multidimentional Assessment of Interoceptive Awareness

mPFC Medial prefrontal cortices

MPA Mixed physical activity

PTF Psychiatric Trauma Research (Psykiatrisk Traumeforskning)

PTSD Post-Traumatic Stress Disorder

RCT Randomised controlled trial

SAE Serious Adverse Event

SAR Serious Adverse Reaction

SCAN Schedules for Clinical Assessment in Neuropsychiatry

SD Standard Deviation

SDS Sheehan Disability Scale

SM Stress Management

SUSAR Suspected Unexpected Serious Adverse Reactions

TAU Treatment as usual

TF-CBT Trauma-Focused Cognitive Behavioural Therapy

VAS Visual Analogue Scale

**1.** **Introduction**

This protocol describes the design of a randomised controlled trial studying two types of physical activity as add-on treatment, compared to treatment as usual for traumatised refugees. The aim is to examine the evidence of an often-used treatment modality in a field where very little research yet has been conducted.

The study will be conducted in accordance with this protocol and current authority requirements/legislation in the area.

**1.1 Background of the trial**

**1.1.1 Traumatised refugees**

The group of traumatised refugees is culturally a mixed group and with a high prevalence of Post Traumatic Stress Disorder (PTSD) and comorbidities to PTSD. A study from 2007 among Danish asylum seekers found 33 countries of origin, where the largest groups came from Afghanistan, Iraq, Iran, Syria and Chechnya (1). Of the 142 examined asylum seekers, 45 % had experienced torture and 63 % of these lived up to the diagnostic criteria for PTSD (1). Moreover, comorbidity of anxiety (2–4), depression (2,3,5) and chronic pain - both somatic and musculoskeletal - (1,6) respectively, has a high prevalence among traumatised refugees with PTSD.

**1.1.1 About the clinic**

The study will be carried out at the Competence Centre for Transcultural Psychiatry (CTP) (before January 2013 named Psychiatric Trauma Clinic for Refugees (PTF)). The clinic was established in 2008 and since 2009 the clinic has been carrying out randomised, controlled trials aiming to increase knowledge about the treatment effect of different types of treatment for traumatised refugees. The target group of the centre is traumatised refugees with PTSD, depression and anxiety, living in the Capital Region of Denmark. Patients are referred to CTP by general practitioners or psychiatrists. CTP treats approximately 200 patients/year.

The treatment is inter-disciplinary and consists of consultations with a medical doctor whereby pharmacological treatment according to best practice in the field is given, supplemented by cognitive behavioural therapy by a psychologist. Manuals are used for both the treatment given by the medical doctor as well as the psychologist. The total duration of treatment is 6 months.

With the aim to increase knowledge about the effect of different types of treatment for multi-traumatised refugees, several randomised clinical trials have been completed in parallel with the treatment since 2009, short after the establishment of the clinic in 2008.

Different manuals have been used for the randomised clinical trials. They have been based on various combinations of Trauma-Focused Cognitive Behavioural Therapy (TF-CBT), Acceptance and Commitment Therapy (ACT), Mindfulness, Stress Management (SM) and Cognitive Restructuring applied in the psychotherapy programme. On the basis of the experience with these manuals, a new manual has been prepared based on cognitive therapy, but adapted to the target group. This manual was used for the last randomised clinical trial and will be used, with minor corrections, in the present study. In the following, therapy given on the basis of this updated manual will be referred to as manual-based cognitive behavioural therapy (manual-based CBT).

**1.4 Previous research**

The decision to initiate the research programmes was taken on the basis of a great need for systematised research in the area, as currently there is a considerable lack of studies on treatment effect in the target group of traumatised refugees. By far the majority of studies of PTSD have been carried out on road victims, rape victims and war veterans. There is reason to believe that the possibility of benefiting from treatment is related to the scope and nature of the trauma that underlies the trauma-related disorder. Therefore, it is a problem that the four Cochrane analyses that exist on treatment effect in PTSD for adults only include very few studies based on refugee populations (7–10).

**1.5 The scientific importance of the present study**

The above-mentioned scarcity of empirical evidence includes a lack of clinical studies of the effects of physical activity as add-on treatment for PTSD.

This is the case both regarding PTSD in the general population as well as PTSD among refugees. Thus, there are still only very few randomised, controlled trials evaluating the use of physical activity as part of the treatment of traumatised refugees (11,12).

According to the public recommendations regarding the treatment of PTSD, among these a NICE-guideline, Cochrane Reviews and a Danish Health Technology Assessment Report, very little evidence is to be found regarding physical activity as part of the treatment for traumatised refugees (7–10,13,14). In the Health Technology Assessment Report from 2008, it is emphasized that neither any international guidelines nor international reviews about the treatment of refugees with PTSD are focusing on the alternative of physiotherapy (13).

Similar to this is the conclusion from the more recent and first Cochrane review made about physical activity as treatment for PTSD from 2010; ”Sports & Games for post-traumatic stress disorder” (7). The conclusion drawn in this is that no studies are fulfilling the inclusion criteria, underlining the limitations of the previous studies in this field, both with regard to quality and the number of studies. Since this review, only a few, new studies have been conducted. Consequently, the basis for giving recommendations about physical activity as treatment for PTSD is very weak (13).

Despite the little evidence, both physical activity in various forms and psycho education regarding physical activity is widely used as an integrated, additive part of the treatment by many Danish institutions treating traumatised refugees (15–18).

Nevertheless, there are several psychological and biological theories as well as clinical studies pointing at how physical activity may have positive effects on other psychiatric illnesses than PTSD (19–41). Among these is depression, an important comorbidities to PTSD (27–32). Positive effects have also been reported for chronic pain, another important comorbidity to PTSD (33–41). A Cochrane Review about the clinical effect of physical activity on depression concludes that it is reasonable to recommend exercise to people with depressive symptoms and to those who fulfil diagnostic criteria for depression (29). However, according to Cochrane, yet not enough evidence exist about: 1) how effective exercise is, 2) the relative benefits of aerobic exercise versus resistance exercise versus mixed exercise, 3) whether group or individual exercises are better, or 4) about the optimum duration of exercise (29).

**1.6 Theoretical framework of the study**

**1.6.1 Physical activity and the physiology**

According to the well-established "Mutual Maintenance Theory" (42) there is a mutual maintenance between the commonly seen comorbidity of chronic pain and PTSD (1,43,44). According to this, treating chronic pain plays a central role in improving the treatment and wellbeing of patients with PTSD.

On a physiological level, studies have shown association between physical activity and a number of biological changes; some which are well known, such as increased muscular strength, improved physical fitness as well as neuro-hormonal changes, e.g. release of serotonin and beta-endorphins (45–48). These could all potentially directly have a positive effect on the mood, or indirectly by stimulating other pathways such as the pathway to neurogenesis in Hippocampus (47,49).

**1.6.2 Physical activity and the autonomous nervous system**

Research shows that with regular physical activity, the part of the nervous system called the autonomous nervous system is affected by increasing the tone of the parasympathetic part. This has been shown to result in a lower resting heart rate and an increased heart rate variability (HRV) (50). Low HRV has been shown to be associated with anxiety and depression (51,52), coronary artery disease and increased mortality (53). High HRV on the other hand is associated with positive emotions (54) and a general resistance to stress (55).

For patients with PTSD, a characteristic pattern has been seen in relation to the autonomous part of the nervous system: PTSD patients have been shown to have a baseline autonomic hyperarousal and lower resting HRV compared to controls, suggesting an increased sympathetic and decreased parasympathetic tone (52,56).

Along with this, studies show that 2/3 of traumatised children and adults have an increased sympathetic activity (57–59).

This characteristic hyperarousal seen in PTSD is, according to van der Kolk, a fundamental dysregulation at brain stem level of the ability to regulate arousal (52). Clinical studies support this, by showing that the baseline heart rate of PTSD patients is significantly increased compared to controls (60–62). Abnormally high baseline heart rate can be either the result of increased sympathetic activity, decreased parasympathetic activity, or both (63). In a clinical study from 2005 (n=59), findings suggest a parasympathetic contribution to baseline heart rate in PTSD and hence supports the belief that poor vagal tone plays a significant role in patients with PTSD (64).

The fundamental dysregulation of the autonomous nervous system in patients with PTSD, which leads to the clinically well-known high level of physiological arousal, makes it important to try to find a way to regulate this.

Studies of magnetic resonance (MR) brain scans have given some knowledge about the functionality of brain structures in patients with PTSD. One part of the brain playing an important role in PTSD is the medial prefrontal cortex (mPFC) which comprises the anterior cingulate cortex (ACC) and medial parts of the orbitofrontal prefrontal cortices (65). The ACC specifically has consistently been shown involved in PTSD and it plays a role in the experiential aspects of emotion, as well as in the integration of emotion and cognition (52,66). A review from 2006 concludes that the ACC is part of a system that coordinates the autonomic, neuroendocrine, and behavioural expression of emotion and may play a key role in the visceral aspects of emotion (66). The mPFC overall has an important role in the extinction of conditioned fear responses through it’s inhibitory influences over the limbic system, whereby it regulates the generalization of fearful behaviour (67).

Bearing in mind the results from neuroimaging studies of people with PTSD showing decreased activation of the mPFC (68,69), and knowing the role that mPFC plays, it has on this background been stated that the dysfunction of the mPFC is likely to contribute to the arousal dysregulation in PTSD (70).

One theory proposes that changing this dysregulation of arousal would be possible through activation of the mPFC and anterior cingulate cortex, by learning to tolerate orienting and focusing the attention on the internal experience, while interweaving and conjoining cognitive, emotional, and sensorimotor elements of the traumatic experience (52).

The fact that the mPFC can directly influence emotional arousal has enormous clinical significance, van der Kolk states. This is suggesting, he says, that activation of interoceptive awareness can enhance control over emotions (52).

This way of approaching PTSD has led to research focusing on activities that improve patient’s awareness on their body and sensations, also called "Body oriented therapies". Among the different types of investigated activities to improve this are for example yoga and Body Awareness Therapy.

Thus, in regard to physical activity as part of the treatment for PTSD, there are many potential, positive effects. Yet there are still a large number of uncertainties regarding e.g. dose, type, form (group/individual), effect size as well as causation of physical activity as treatment.

**1.6.3 Basic Body Awareness Therapy**

One type of physical activity that in the last decades has been evaluated in a number of trials on different illnesses is Basic Body Awareness Therapy (Basic BAT), a modification of BAT (Body Awareness Therapy), which was first described by the French psychoanalyst and actor, J. Dropsy. In 1985, the Swedish physiotherapist G. Roxendal started using BAT in patients with schizophrenia (71).

Basic BAT focuses on the basic function of movements related to posture, coordination, free breathing and awareness (72) and aims to normalize posture, balance and muscular tension, which are experienced and visible in the movement pattern (39).

Basic BAT is a body-oriented physiotherapeutic approach. BBAT uses a perspective on physiotherapy treatment directed towards an awareness of how the body is used, in terms of body function, behaviour and interaction with the self and others (72).

BAT and Basic BAT has shown positive effects in regard to a number of psycho-social factors on several groups of patients, some of which share common problems with traumatised refugees, such as patients with non-specific musculoskeletal pain and chronic pain (38,39,73).

In a study from 2001, BAT, Feldenkrais and conventional physiotherapy have been compared regarding psychological distress, pain and self-image in patients with non-specific musculoskeletal disorders (37,38). All three groups improved in most of the health survey dimensions; particularly physical function, pain, general health, vitality, social function, emotional function and psychosocial health. There were no significant differences between the groups although the BBAT group tended to improve more in terms of physical function and general health than the other two groups. Few significant differences were seen but larger effect sizes in improvements were shown in the BAT- and Feldenkrais-groups for somatisation, anxiety, depression and self-image as well as improvement of bodily pain compared to standard physiotherapy (37,38).

Another study was carried out on patients with somatoform illness, mood changes and personality disorders (19). This study shows a significant effect on physical coping-resources, sleep-quality and body awareness, which are all key issues for traumatised refugees. Moreover, an improvement in body awareness, approach to the body as well as self-efficacy, sleep and physical coping resources compared to standard treatment are mentioned as benefits (13). Regarding the degree of chronic pain, this group is by the Health Technology Assessment Report (HTA) asessed comparable to refugees with PTSD (13). Body Awareness Therapy is thus mentioned as a concrete example of a physical activity with potential benefits on the group of traumatised refugees. However, the above mentioned studies were all carried out on other groups of patients – most with a non-traumatic background – with different medical and mental problems than the population of traumatised refugees. Both regarding their mental and physical problems, social situation and cultural background respectively – the HTA report emphasizes that further research in the different types of physical activities should be carried out on the specific population of traumatised refugees (13).

In the light of this it becomes clear that there is a big need for more and well-conducted clinical trials on this specific group of patients if scientific evidence should be the basis for recommendations on whether physical activity should be part of the treatment for traumatised refugees.

**1.6.4 Study of the feasibility of Basic BAT for traumatised refugees**

In order to test the feasibility of Basic Body Awareness Therapy (Basic BAT) on a group of traumatised refugees, a pilot project was carried out in 2012 (data has not yet been published) at the Competence Centre for Transcultural Psychiatry. In the pilot project, a group of Arabic-speaking patients were offered treatment with Basic BAT, in groups once a week during 13 weeks. Fourteen patients were included and 9 patients continued throughout the whole treatment period (5 men and 4 women). Due to the small sample size of the study, the data were primarily analysed qualitatively, in terms of semi-structured interviews conducted with all patients. Some patients decided not to participate in the study due to the group element, and some afterwards said they had found the intervention period too short. Overall though, patients showed a high acceptability and compliance to the type of physical activity and a high satisfaction with the treatment, as it was also shown in an earlier study (73). The clinical impressions of the physiotherapists were that the focus of Basic BAT on gentle body awareness was highly beneficial for these patients, because of their degree of chronic, bodily pain and therefore long-time mental disconnection from the body or bodily dissociation (74–77).

Meanwhile, in order to show actual evidence of this treatment, these results need replication in a bigger study. The positive results of this pilot study are part of the basis for the present research project, but these results need replication in a larger study.

**1.6.5 Mixed physical activity as intervention for traumatised refugees**

Among the few randomised controlled trials made on traumatised refugees with PTSD, with physical activity as intervention is one study published after the Cochrane review (29) It is a smaller study by Liedl et al. (11). It is based on the well-established "Mutual Maintenance Theory" (42), which suggests the causation between PTSD and the commonly seen co-morbidity chronic pain (1), as being based on mutual maintenance (42), In the study the effects of mixed physical activities (exercises of stretching, strength and endurance) as additive therapy to psycho education and biofeedback-based cognitive behavioural therapy (CBT-BF) was examined. The study showed improved coping strategies, larger effect sizes, and higher rates of clinical improvement in the physically active group, suggesting that physical activity adds value especially to pain management for traumatised refugees (11). Still, the study is small (n= 30) and these preliminary results need replication in a larger trial (11).

**1.7 The present study**

Hence, the background for the present study is 1) The general literature on the field, 2) The above-mentioned studies of Basic Body Awareness Therapy on groups of patients that, in terms of certain common characteristics in symptoms, have some comparability to traumatised refugees, and 3) The above-mentioned study of mixed physical activities on traumatised refugees

The study will in a larger patient group of 200 patients examine the positive but preliminary add-on effects of these two different types of physical activity when added to treatment as usual. This will contribute to important knowledge that can be added to the present scarce amount of evidence. The aim of this is to facilitate better, future treatment for traumatised refugees and thereby improve mental and physical health for these patients.

The effects will be measured in terms of improvement of PTSD, depression, anxiety, quality of life, and functional capacity. In addition coping with pain, body awareness, physical strength, endurance, balance and coordination will be assessed.

**1.8 Aims of the study**

1. *To examine whether physical activity as an add-on treatment to psychiatric treatment as usual, gives an increased effect compared to psychiatric treatment as usual in mental symptoms (PTSD, depression and anxiety), quality of life, functional capacity, coping with pain and body awareness.*
2. *To examine, whether an increase in physiological parameters such as strength, endurance, balance and coordination correlates with an improvement of PTSD, anxiety, depression, coping with pain, quality of life, functional capacity as well as body awareness and, respectively.*
3. *To examine if the number of hours spent on home exercises in the specific assigned physical activity is a positive predictor of the treatment effect.*
4. *To examine if BBAT has a higher impact on mental symptoms (PTSD, depression, anxiety), coping with pain, quality of life, functional capacity, and body awareness, compared to mixed physical activity.*

**1.9 Hypotheses**

1. *Physical activity as an add-on treatment to psychiatric treatment as usual, has an increased effect compared to psychiatric treatment as usual with respect to mental symptoms (PTSD, depression, and anxiety), coping with pain, quality of life as well as functional capacity and body awareness.*
2. Increase in physiological parameters such as strength, endurance, balance and coordination is positively associated with improvement in PTSD, depression, coping with pain, quality of life, functional capacity as well as body awareness respectively.
3. *The number of hours spent on home exercises in the specific assigned physical activity is a positive predictor of the treatment effect.*
4. *BBAT has a larger impact on mental symptoms (PTSD, depression, anxiety), quality of life, functional capacity, coping with pain and body awareness, compared to mixed physical activity.*

**2.** **Research methods**

**2.1 Trial design**

Number of patients (n): over a period of about one year and eight months 250 patients will be included by the clinic’s five-six doctors (see also annex 1 for the study design). Previously randomised projects in the clinic (notified to The National Committee on Health Research Ethics under H-D- 2009-038 and H-D-2011-020 and H-3-2012-020) have included about 150 patients per year, and 75-80% of these patients have completed the project. Therefore 250 project patients are deemed realistic within the given time frame. All patients are invited for a referral interview (see an example of an invitation to this interview attached to this protocol called “Indkaldelsesbrev”) to clarify whether the patient meets the inclusion and exclusion criteria, and if so, and if the patient gives her/his consent to participate, the patient is randomised to one of the three intervention groups:

The three groups:

Group 1) Control group: receives treatment as usual (TAU) for six month, which consists of: consultations and medical treatment according to best clinical practice in the field by a medical doctor as well as manual-based CBT by a psychologist. The medical consultations run for six months. The first two months includes medication and psycho-education approximately once a week followed by another four months of one monthly consultation with a doctor to adjust medication etc. and manual-based CBT two to four times per month (for a description of CBT, see later chapter). Henceforth, the treatment just described for the control group is denoted as treatment as usual (TAU).

Group 2) Basic Body Awareness Therapy (BBAT): Treatment as usual as described in the section above and approximately 2 weeks after the beginning of treatment starts the individually based BBAT alongside treatment as usual. The Basic Body Awareness Therapy will proceed for 20 weeks with one weekly training session, each session being approximately 1 hour long. For a more detailed description of BBAT, see the later section on this.

Group 3) Mixed physical activity (MPA): Treatment as usual as described in the section above and approximately 2 weeks after the beginning of treatment starts the individually based MPA alongside treatment as usual. The MPA will proceed for 20 weeks, with one weekly training session, each session being approximately 1 hour long. For a more detailed description of MPA, see the later section on this.

Course of treatment and data collection will follow the CONSORT statement (78).

For a more detailed description of the course of treatment, see below.

In all three groups, after the referral interview and a preliminary psychological assessment, the referring doctor and the psychologist will carry out an assessment of the expected outcome, and on the basis of this assessment categorise the patients into three categories: High, moderate and limited expected outcome. At the end of the treatment period, the patients will be placed in one of the three groups again, but this time according to actual outcome measured as improvement in percentage of scores on the HAM-D and A.

**2.2 Inclusion criteria:**

- Patients referred to treatment for PTSD at Competence Centre for Transcultural Psychiatry from September 2013 to May 2015.
- Adults (18 years or older)
- Refugees or persons who have been brought in under family reunification
- Symptoms of PTSD pursuant to the ICD-10 research criteria.
- Psychological trauma in the anamnesis. Trauma is typically imprisonment or detention with torture (according to the UN definition of torture) or acts of cruel, inhuman and degrading treatment or punishment. Trauma can also be organised violence, long-term political persecution and harassment, or war and civil war experiences.
- Assessed by a doctor to be motivated for treatment
- Informed consent.

**2.3 Exclusion criteria**

- Severe psychotic disorder (defined as patients with an ICD-10 diagnosis F2x and F30.1-F30.9). Patients are excluded only if the psychotic-like experiences are assessed to be part of an independent psychotic disorder and not part of a severe PTSD and/or depression.
- Current abuser of drugs and alcohol (F1x.24-F1x.26).
- In need of admission to psychiatric hospital.
- No informed consent.
- Physical handicaps that make the person unable to participate in the physical activity.
- Cardiac arrhythmia identified on the electrocardiogram taken before start of the treatment or symptoms of heart problems that are unclarified.

**2.4 Outcome measures**

Primary outcome measures:

Harvard Trauma Questionnaire (HTQ) (79).

Secondary outcome measures:

Hopkins Symptom Check List (HSCL-25) (80), HAM D+A (81), WHO-5 (82), GAF-S & GAF-F (83), Sheehan Disability Scale (SDS) (85), Pain on Visual Analogue Scale (VAS) (87), Brief Pain Inventory short form (BPI) (88,89), Multidimensional Assessment of Interoceptive Awareness (MAIA) (90). Test for physical parameters: Tandem Gait Test and Single-leg stance test, Dynamic Gait Index (DGI) (91), Senior Fitness Test (SFT) (92), De Morton Mobility Index (DEMMI) (93,94).

**2.5 Randomisation**

All patients will be randomised after a two-hour referral interview with a doctor in accordance with inclusion and exclusion criteria. Randomisation is carried out by the Department of Biostatistics at the University of Copenhagen, with practical assistance from a group of secretaries not linked to the daily work at CTP. Stratification by gender and level of severity of PTSD symptoms on the basis of HTQ will be carried out before randomisation.

The actual randomisation is carried out as follows: the Department of Biostatistics draws up a randomisation list of which patients are receiving which treatment (anonymous). A person who is not associated with patient treatment will convert this randomisation list into numbered envelopes. The envelopes will be supplemented by a list of what envelope is given to what patient (patients are identified by a number and not by name). The randomisation list will be kept at the Department of Biostatistics, and a secretary from the Psychiatric Centre Ballerup, who is in charge of the practical randomisation, also holds a copy. After each referral, the referring doctor will contact the secretary mentioned above if the patient is to be included in the trial. The secretary opens the envelope and informs the doctor about which intervention group the patient will be in. This method has been applied in all previously randomised projects at CTP, has been approved by the Ethics Committee of the Capital Region of Denmark and has proven to be satisfactory in practice.

**2.6 Blinding**

Blinding patients is obviously not possible and blinding doctors and psychologists is not deemed to be possible due to the many consultations during the period in which the patient is practicing physical activity. Unavoidably there will in the consultations be shared some information about how the physical activity is going and what type of activity is being practised. However, an intervention-group blinded HAM D+A rating will be carried out at the beginning and at the end of treatment. Hamilton raters will be trained at the clinic and will take part in regular joint ratings to ensure high quality and interrater reliability.

**2.7 Drop-out**

On the basis of the completion rate in previous similar randomised trials at CTP, 75-80% of the patients are estimated to complete the treatment.

**2.8 Extraction criteria**

Patients will typically be eliminated from the trial, if they have failed to show up more than three times in a row. Patients will be eliminated, if they have been assessed to be suicidal or psychotic to a degree that requires acute admission during the treatment period. Patients will also be eliminated if investigator is assessing that the treatment is directly damaging the health of the patient. Furthermore, patients that do not wish to continue in the study will be eliminated.

**2.9 Programme fidelity**

In order to determine programme fidelity, the psycho-education topics addressed will be registered at the end of each consultation with the medical doctor. It will also be registered whether the main content of the consultation was of social, psychological or somatic character. After each psychologist consultation, the topics addressed will be registered in the patient records, as well as the methods used during the consultation and whether the patient has completed his/her homework as planned. Patient attendance will be registered, and at each consultation with a medical doctor, patients will be asked if they have taken their medication as agreed. Medication accounts will be kept to assess pharmacological compliance. Patients will be included in the data set despite poor compliance.

The physical activity for each respective group will follow a manual developed for that specific type of physical activity (BBAT and CPA respectively).

At every session of physical activity, the physiotherapist will register the attendance of each patient and his or her individual degree of participation in each exercise.

The two types of physical activities will be administered independently and parallel to consultations with the medical doctor and the psychologist.

**2.10 Representativity**

Patients have not been selected on the basis of more specific criteria than elsewhere in the treatment system, and therefore are similar to the population of patients found at a clinic treating trauma-related disorders in refugees. Thereby, the results can be generalised to other corresponding patient groups and are directly applicable in the planning of treatment.

**3.** **Course of treatment**

The treatment consists of the treatment as usual (TAU) the way it is normally carried out in the clinic and as described above, combined with physical activity as an add-on treatment. The two types of physical activity are respectively Basic Body Awareness Therapy (BBAT) and mixed physical activity (CPA).

Regardless of intervention group, treatment as usual is given for a total of six months and can be extended by a maximum of one month.

The physical activity will be running parallel to the treatment as usual once a week for 1 hour per training session and for a total of 20 times of training.

All patients will be motivated to do homework in terms of home exercises from the respective type of physical activity, shown in the sessions of physical activity.

For description of treatment as usual, see section above.

**3.1 Referral**

Patients are referred to the clinic by their general practitioner (GP), a private practice psychiatrist or by doctors from psychiatric centres. The senior doctors at CTP go through all referrals, and on the basis of the referral, patients are invited for a referral interview. If it is clear from the referral that the patient does not belong to the clinic’s target group, the patient is not invited for a referral interview.

**3.2 Referral interview**

The referral interview takes about two-three hours and consists of a clinical assessment, recording of anamnesis, information about the treatment and the trial as well as the first patient rating. An electrocardiogram will also be recorded. If the patient consents to participate, he/she will be randomised after the referral interview to one of the three groups. Standardised diagnostic tools such as SCAN and the ICD research criteria will be applied in the referral interview.

**3.3 First psychological assessment**

Before the treatment by the psychologist begins, a preliminary psychological assessment is carried out in order to assess whether the therapy is appropriate for the patient. Moreover, in consultation with the referring doctor, expected treatment outcome is assessed and the patient is placed in one of the three expected outcome groups as described above.

**3.4 Physical activity**

All patients will be randomised into one of three groups:

1) Control group (treatment as usual and psychotherapy as described in the manual)

2) Body Awareness Therapy

3) Mixed physical activity

For detailed descriptions of the interventions, see the separate sections on this below.

**3.5 Medical treatment**

The medical treatment (part of the treatment as usual) consists of a total of ten consultations of about 45 minutes. During the first six weeks, the patients will see a medical doctor once a week, and over the next four months about once a month. The first consultation will typically take place one-two weeks after the referral interview. In addition to the pharmacological treatment, patients will receive psycho-education in an individually adjusted course, in which relevant topics will be addressed such as knowledge of disease (PTSD, depression and anxiety), pain, sleep, concentration and memory as well as exercise and lifestyle. The consultations with the medical doctor are described in a manual that is followed by all doctors.

**3.6 Psychologist consultations**

A psychologist carries out psychotherapy (part of treatment as usual) over 16 consultations of 45 minutes stretched over a period of four months according to the CBT manual as described above. Psychologists who have all been trained in using this manual carry out the psychotherapeutic treatment.

**3.7 Social counsellor**

At the beginning of the treatment course, all patients will be offered an appointment with a social counsellor (part of treatment as usual) to clarify their social situation, and a second appointment is offered at the end of the treatment course. During the course of treatment, patients can receive additional assistance from a social counsellor if required. This offer is individualised. All social counsellor services will be recorded. Other services related to social functioning, for example establishing contact to social programmes for the target group, will also be registered in the patient records.

**3.8 Evaluation and follow-up**

All courses of treatment will be completed with an evaluation interview attended by patient, medical doctor, psychologist and, if necessary, social counsellor. In addition to this, the patient will fill in a questionnaire for the satisfaction study. All patients will be invited to take part in a follow-up interview that includes ratings about six months and 18 months after completed treatment.

**3.9 The patient group**

Patients with war-related trauma have certain characteristics, which might challenge the completion of the trial. The majority of the patients taking part only speak very little Danish, and an interpreter is present during 60-70% of the interviews. Most patients are expected to come from Afghanistan, the former Yugoslavia, Palestine, Russian-speaking countries, Iran, Iraq, or from other Arabic-speaking countries in the Middle East. Many of the patients have no or very little education, which means some are illiterate.

As a consequence of their psychiatric disorder, many patients have cognitive problems such as attention deficit, problems with memory and concentration. Many also suffer from anxiety, which can be disabling with regard to social function. This affects attendance and compliance. Finally, the psychological problems of the patients are often expressed as somatisation and complaints about pain apparently without underlying physical disorder.

**3.10 Interpretation**

Translated versions of all rating scales are used, and these will be translated into the five main languages of the clinic (Danish, English, Arabic, Bosnian and Farsi). Interpreters will be always be available when needed and present at ratings as required. The interpreters are all affiliated with the CTP and are experienced in interpreting questionnaires, psychotherapy and psycho-education consultations.

**3.11 Variability in the treatment programme**

As far as possible, all patients will follow the treatment model prescribed, but there will be some variation in attendance, as the patients may become ill, or for some other reason not show up for consultations (please see the problems with compliance described above.)

**3.12 Treatment responsibility**

While the patient is receiving treatment at CTP, the attending medical doctor at CTP is overall responsible for treating the patient for the mental health problems. The patient’s GP is still responsible for treating somatic disorders in collaboration with any somatic medical specialists in the primary or secondary sectors. As long as the patient is being treated at CTP, treatment by any private practice psychiatrist and/or psychologist will be paused.

**3.13 Collaboration with the primary sector**

Good collaboration with the patient’s GP is vital in order to provide the best possible treatment for the patient. CTP sends a letter to the GP at the beginning of treatment and at the end of treatment, and as required during the treatment.

**4.** **The physical activities:**

All patients taking part in PTF4 will be given treatment as usual alone or with either BBAT or mixed physical activity. Monitoring of the effect on physical fitness during the training will take place for participants of both Basic Body Awareness Therapy and mixed physical activity: an objective based physical performance test battery will be carried out by the physiotherapist in the beginning and in the end of the period of training, quantifying upper and lower extremity function in regards to strength, endurance, balance and coordination. All patients will be told to pause the physical activity if they experience any discomfort that can be related to heart problems. To evaluate the effect of physical activity in relation to body awareness and coping with pain respectively, self-rating scales in the form of Pain Inventory and Multidimensional Assessment of Interoceptive Awareness (MAIA) will be used. These self-ratings will take place in the beginning and at the end of the 20 weeks of assigned physical activity.

To evaluate the effect of physical activity in relation to physical parameters of fitness (strength, endurance, balance and coordination), an individually based objective test battery in the form of Senior Fitness Test (SFT), De Morton Mobility Index (DEMMI), Dynamic Gait Index (DGI), Semitandem Stand, Full Tandem Stand and Single-leg Stance Test will be used. These tests will take place in the beginning and at the end of the 20 weeks of physical activity.

At each session, the physiotherapist will record the extent to which the patient was able to do the physical exercises in the session and the amount of reported physical activity homework the patient has done since last session.

**4.1 Basic Body Awareness Therapy (BBAT)**

The teaching of BBAT will be carried out by physiotherapist who are certified BBAT-trainers. The participants of the study will be scheduled to one weekly, individual BBAT sessions for a total of 20 weeks. One BBAT session lasts approximately 1 hour and an interpreter will be present at each session. At the weekly BBAT session the physiotherapists will register the attendance of each patient and his or her individual degree of participation in each exercise.

**4.2 Mixed physical Activity (MPA)**

Physiotherapist that are used to working with traumatised refugees have developed the manual for the teaching in MPA, and likewise will physiotherapist who are used to work with traumatised refugees carry out the teaching of the MPA. The participants will be scheduled to one weekly, individual BBAT sessions for a total of 20 weeks. One MPA session lasts approximately 1 hour and an interpreter will be present at each session. At the weekly MPA session the physiotherapist will register the attendance of each patient and his or her individual degree of participation in each exercise.

**4.3 Home exercises**

For both assigned physical activities the physiotherapist will motivate the patients to do home exercises as much as possible. The homework will be specific exercises from the type of physical activity that the patient is assigned to and will be demonstrated in the training sessions. The physiotherapist will motivate the patients to record in a diary given for the same purpose the amount of time spent on homework and the type of exercise. After every session with the patient, the physiotherapist records these details about the homework in the patient's files. The patients will be given exercise mats to use at home as to make it easier to do the home exercises. In order to be able to continue with the physical activity after the course of treatment, the patient is offered to keep the mat.

- 1. **Adverse events**

All adverse events will be registered in the individual patient records in the period from the first consultation at the centre to the last consultation at the centre of each patient*.*

All Serious Adverse Events (SAEs) will be reported by sponsor or investigator to The National Committee on Health Research Ethics (within 7 days after investigator has become aware of any such), with comments enclosed on potential consequences for the trial.

Sponsor will yearly submit a report to The National Committee on Health Research Ethics on all SAEs, with an assessment attached on the security of the patients participating in the trial.

The report will be submitted each year on the day when the trial was given approval from The National Committee on Health Research Ethics.

Participation in the study is not expected to course any SAEs. Expected adverse events would be in the form of the potential muscular discomfort which using the muscles can lead to and a general bodily tiredness from using the muscles. The assigned physical activity is beside this mainly expected to give an extra, positive element to treatment as usual, that the patients normally would not get.

**5.** **Data collection**

Both self-ratings and observer-ratings will be used in the project.

**5.1 Rating scales**

The following rating scales (available in Danish, Arabic, English, Croatian and Farsi) will be applied:

- **The Harvard Trauma Questionnaire (HTQ)** (95) is an internationally applied and thoroughly validated self-administered rating scale assessing the severity of PTSD symptoms. The first 16 questions of the HTQ, Part IV (symptoms part) are used to monitor PTSD symptoms. These 16 questions cover all PTSD criteria in accordance with ICD-10 as well as DSM-IV.
- **Hopkins Symptom Check List (HSCL-25)** (80) is an internationally applied and thoroughly validated self-administered rating scale assessing the severity of anxiety and depression symptoms. This is a short version of the Symptom Checklist -90 (SCL-90). It consists of 25 questions, 10 regarding anxiety and 15 regarding depression. In addition to this, the somatisation part of SCL-90 is used.
- **WHO-5** (82) is a self-administered questionnaire evaluating quality of life, and it consists of five questions with six possible answers. The questionnaire has been used to assess the quality of life in a number of psychiatric diagnosis groups. In addition, the scale has been used to assess overall treatment effects in the field of psychiatry.
- **Sheehan Disability Scale (SDS)** (85) is a self-administered rating scale measuring functional impairment with regard to family, work and social network using three visual analogue scales from 0-10. Evaluation of the scale has shown that it is sensitive to treatment effects in psychiatric patients.
- **GAF-S & GAF-F** (83) are numeric observer rating scales used to assess the degree of social and physical functioning in adults. The scales are widely used in the field of psychiatry. They have been validated in many languages and are used frequently in clinical studies in the field. They have been applied in studies on PTSD in a number of patient groups.
- **Pain on Visual Analogue Scale (VAS)** (87) Patients’ pain will be assessed in connection with ratings, assessing pain intensity in the head, back, upper extremities and lower extremities with visual analogue scales (VAS)
- **Hamilton depression and anxiety scales (HAM-A and HAM-D)** (81) are observer rating scales assessing depression on the basis of semi-structured interviews. The scales have been used for psychiatric research and assessment of symptoms in torture survivors. They do not require translation as practitioners themselves formulate the questions on the basis of a multiple-choice form.
- **Brief Pain Inventory short form (BPI)** (88) is a self-report instrument assessing severity of pain, impact of pain on daily function, location of pain, pain medications and amount of pain relief in the past 24 hours or the past week. Psychometric and linguistically validated on more than 20 languages. Cronbach alpha reliability between 0.77 and 0.91. Developed by the Pain Research Group of the WHO Collaborating Centre for Symptom Evaluation in Cancer Care.
- **Multidimensional Assessment of Interoceptive Awareness (MAIA)** (90) is a self-report instrument, developed by E. Mehling in 2012 to assess interoceptive body awareness.
- **Semitandem stand, Full Tandem Stand and Single-leg stance test** is testing the static balance.
- **Dynamic Gait Index (DGI)** (91) is developed by Shumway-Cook in 1995 to evaluate on the dynamic/functional balance.
- **Senior Fitness Test (SFT)** (92) is an objective test of function consisting of six parts assessing upper and lower body strength, upper and lower body flexibility, aerobic fitness, and agility strength.
- **De Morton Mobility Index (DEMMI)** (93,94) was developed to assess the mobility of patients with many physical limitations.

**5.2 Frequency of rating**

Patients will be asked to complete self-ratings three times during the course of treatment: at the referral interview, before the first psychotherapy session and at the end of the treatment course. Blinded Hamilton rating will also take place in connection with start of the six months of treatment and completion. The objective physical fitness tests and the self-ratings in the form of Pain Inventory Short Form and Multidimensional Assessment of Interoceptive Awareness (MAIA) will take place at the beginning and at the end of the 20 weeks of assigned physical activity. See also flowchart in annex 2.

**5.3 Risk factors**

To explore the participants’ motivation, previous body experiences, eventual barriers to participate, and their satisfaction with and experienced effects of BBAT and mixed physical activity, semi-structured interviews will be conducted at the start and at the end of the period of physical activity.

At each consultation with a doctor, the patient’s condition will be assessed at a standardised psychiatric examination covering 14 parameters. In addition to the above, the severity of the disease will be assessed at the referral interview and at the regular ratings described above. The patient’s ability to engage in psychotherapy will be assessed by the attending psychologist before treatment is started and again once during the course of treatment.

During consultations with the doctor and psychologist, the doctor/psychologist will morally support the patient in attending the physical activity. He or she will discuss with the patient adherence to the physical activity program and help identifying potential obstacles to the training and, if possible, help to overcome the obstacles

**5.4 Satisfaction study**

The satisfaction study will be conducted after completion of treatment. After the evaluation interview, patients will be asked to fill in a questionnaire on patient satisfaction, if necessary assisted by an interpreter. This is standard procedure in the treatment at CTP and is not specific for this project. The satisfaction questionnaire is anonymous, as only the patient’s ID and not his/her civil registration number is written on the form. The group of practitioners will not have access to the individual patient questionnaires.

**5.5 Drop-out study**

All patients who do not complete the treatment will be asked about their reasons for not completing treatment. This as well as noting when during the course of treatment the dropout has happened will be registered in the patient records.

**6.** **Safety, quality assurance and responsibility**

**6.1 Responsible for data collection**

The investigator is overall responsible for data collection. The doctor responsible for treatment is also responsible for data collection from his/her patients. All ratings will be carried out by a doctor or in the presence of a doctor (for self-ratings) or a physiotherapist (for self-ratings). A doctor, a psychologist, a physiotherapist as well as a social counsellor will carry out collecting data on risk factors/background information. A research assistant or an investigator will carry out the satisfaction study and dropout study. Blinded observer ratings (Hamilton rating) will be carried out by a doctor or a medical student not linked to the team of practitioners, but to an independent Hamilton team with thorough training in using the rating scales used in the project. In order to ensure quality and inter-rater reliability, the members in the Hamilton team participate regularly in joint ratings under the guidance of one of the permanent doctors at CTP.

**6.2 Data security**

All data collected for this project will be protected according to the Act on Processing of Personal Data as well as the Danish Health Act. In order to ensure satisfactory inspection and monitoring of the trial by relevant bodies, the investigator will allow direct access to source data/documents (including patient records) in connection with monitoring, auditing and/or inspection by the GCP unit.

**6.3 Source data**

All data registered on the patients will be available as source data in the form of original rating forms completed by the patients or practitioners, as well as structured patient records. Data will be saved for 15 years after the trial has ended, which will be stated in a letter of attorney signed by the patients. If Case Report Form is source data, this will be described in the source data document, which will be filed in the Trial Master File.

**6.4 Quality assurance**

Quality control and quality assurance will follow regular procedures as described in sections 3 and 4 of the Danish Executive Order on good clinical practice (GCP). Though it is not mandatory for this trial to be monitored by GCP, this has been chosen in order to assure the highest possible quality of the study. The trial will be notified to the Danish Data Protection Agency through the Capital Region of Denmark. Managing and filing data will be in accordance with current guidelines for research. No information from previous patient records will be used directly in the trial, but the attending doctor will be familiar with information from referrals and included record copies. In order to ensure interrater reliability, all doctors will attend a SCAN course, and regular joint ratings of the patients will be carried out with clinical assessment and GAF.

**6.5 Treatment responsibility after the trial**

At the end of the trial, the patients will be referred to follow-up consultations and any necessary treatment by their own GP, private practice psychiatrist (perhaps the same psychiatrist who referred the patient) or at the community psychiatry centre. Patients who drop out before planned will be treated by their own GP. The GP will receive written information on the treatment completed and individual recommendations for future treatment based on the attending doctor’s knowledge of the patient.

**7.** **Data analysis**

**7.1 Size of material**

The trial will be stopped when 65 patients in each group have completed treatment; that is when a total of 200 patients have completed the trial. The estimated drop-out rate is about 25%, which means that about 250 patients will have to be included in the trial.

**7.2 Drop-out analysis**

Drop-out analysis is based on the patients who show up at the initial referral interview. The patients in the programme will be compared with the patients who were excluded at the referral interview in order to identify possible systematic selection bias. Furthermore, the group of patients included in the trial, but who eventually drop out and do not complete the trial will be analysed. In addition to completer analysis intention-to-treat analyses will be carried out.

**7.3.** **Power calculations**

If 200 patients are divided into three groups of about 65 patients, power to detect a group difference in the treatment effect of ½ SD will be 81%, while power to detect a difference of 1 SD will be close to 100%. Differences in quantitative outcomes less than ½ SD between the two treatments are considered to be less relevant from a clinical point of view. Cut-offs are available for several rating scales, and these can be used to define categorical outcome variables. If for example the proportions below cut-off for clinical case status are 50% and 25% in two groups, power will be close to 80% to detect a significant group differences.

**7.4. Data processing**

The primary outcome variables are changes during the treatment course calculated as differences between start, middle and end ratings. The unadjusted differences between the three trial groups can be analysed by one-way ANOVA and pairwise comparison of means, while adjustment of the differences for baseline values and possible background factors (such as gender and age) can be done by ANCOVA/linear regression.

In addition, cut-off can be used on symptom scores and logistic regression analyses can be carried out with over/under cut-off as binary outcome. In addition to completer analyses, drop-out analysis and intention-to-treat analyses will be carried out.

Hypotheses concerning predictors of treatment effects can be analysed by including the potential predictors in linear or logistic regression models including treatment group and relevant covariates.

**8.** **Ethical considerations**

As mentioned previously, there is not enough scientific knowledge about the treatment of traumatised refugees, neither regarding physical activity as treatment, nor medical nor psychological treatment. Therefore, randomised, controlled effect studies in this area are needed. This implies that results of this trial are expected to improve treatment for the individual patient and to stimulate to further research within a relatively short time. In the long term, results are expected to be applied in reference programmes and clinical guidelines. This will ensure patients well-proven treatment of high quality as well as ease the work of practitioners with regard to finding the right type of treatment for the patient. Furthermore, the trial is expected to generate a socio-economic gain, as it will prevent expensive treatment that does not work.

No serious disadvantages or serious adverse events (SAEs) are expected in any of the groups of the trial.

The two types of physical activities are very gentle which is why it is expected to give, if any, only mild side effects. Like it is mentioned earlier, expected side effects would be in the form of the potential muscular discomfort which using the muscles can lead to and a general bodily tiredness from using the muscles. The assigned physical activity is beside this mainly expected to give an extra, positive element to treatment as usual, that the patients normally would not get.

Cognitive behavioural therapy methods have been applied since CTP opened, and these methods have been adapted continuously to the target group of the clinic.

This means that patients taking part in the trial will be given the treatment assessed to be the most optimal on the basis of the evidence available, and hopefully the trial will also generate new knowledge to the benefit of future patients.

The protocol will be submitted to the Ethics Committee for approval. The project recognises the Declaration of Helsinki II.

**8.1 Informed consent**

Taking part in the trial is voluntary and requires written informed consent. Patients can stop treatment and leave the trial at any time. Taking part in the trial is not a prerequisite for receiving treatment at the clinic. Participants will be informed about the trial verbally and in writing. They can obtain further information from the doctor responsible for the treatment or the person responsible for the project (investigator) at any time.

Written information about the trial will be sent to the patient with an invitation to a referral interview. This information will typically be sent two weeks before the referral interview. The letter states that research will be carried out CTP, and that the patient will be informed in more detail about this at the interview. Moreover, patients will be informed that they can bring a relative or a friend to all of or some of the interview. The written information about the trial will be available in five languages: Arabic, Farsi, English, Bosnian and Danish. These languages cover more than 90% of the languages spoken at the clinic. At the referral interview, patients will be asked whether they have received and read the material sent to them. If they have not been able to read and understand the material, for example due to illiteracy, the written material will be read aloud by the interpreter and explained in detail to make sure the patient fully understands the material.

Oral information about the trial will be given to the patients at the referral interview, which is also the patient’s first contact to CTP. At the referral interview, the doctor will assess whether the patient falls within the target group of the clinic. If so, the patient will be offered treatment. Subsequently, the patient will be informed orally about the study in accordance with these guidelines. If required, a professional interpreter, who will be present throughout the entire referral interview, will translate the information.

The oral information states that CTP applies psychotherapy by psychologists and supporting discussions as well as medication administered by doctors, and that all patients will undergo a combination of these two treatment options at the clinic. Patients will be informed that the effects of the two physical activity interventions are unknown and that it is therefore important to conduct a study of this nature. Information will also include that the treatment methods applied are associated with very few potential side effects and risks. Immediately after this oral presentation of information, the written information will be explained. The written information will be read aloud by the interpreter and the patient is given the opportunity to ask any questions he/she might have. Referral interviews will take place in undisturbed surroundings at the doctor’s office. Sometimes patients will bring relatives to the referral interviews. As the referral interview involves questions about the patient’s trauma history, relatives are not normally recommended to be present during this part of the interview. However, if the patient wants a relative to be present, this can be arranged.

The patient will be encouraged to sign a declaration of consent in connection with the referral interview. If the patient needs more time to think before signing the declaration of consent, this will be possible. After the patient has given written consent, he/she will be randomised to an intervention group and be informed about the time and date of the next consultation and about the content of the course of treatment.

Whenever the patient might like to have more information about the project, information is available through the medical doctor conducting the referral interview. This doctor is also the person responsible for the treatment throughout the course of treatment.

At any time patients can end their treatment or their participation in the trial without consequences for their treatment.

**8.2 Ethical aspects in randomisation**

Randomisation is assessed to be ethical, as there is limited knowledge about the treatment effect in the patient group, and as there is insufficient knowledge about which of the treatments offered to the patients is the best. Moreover, all patients will be given treatment as part of the trial. In addition to the above, it would be unethical to continue to give patients long-term and expensive treatment, if the effect of it is not known. Due to the very limited evidence in this area, this would be the case, if no attempts were made to create evidence on the treatment effect.

Those patients included in the trial but not randomised to physical activity will, according to the medical doctor’s assessment, be offered to participate in mixed physical activity after the end of treatment course.

**8.3 Right of access to patient records**

Participants can be given access to their own data after the trial to the extent this is possible without compromising confidential information about other patients.

**8.4 Disadvantages of the trial**

No serious disadvantages or side effects are expected in any of the treatment options applied. Patients will fill in self-ratings during the trial. However, other than this, there are no inconveniences. These ratings have been used as an evaluation tool in the normal clinical practice at CTP since it was established in 2008, and have not previously been seen as problematic by the patients. Surgical procedures on the patients or removal of biological material will not take place.

**8.5 Insurance and compensation**

The current patient insurance programme of the Capital Region of Denmark - Psychiatry, protects the patients.

**9.** **Project organisation**

**9.1 Project initiation**

The project has been initiated and developed in cooperation between Investigator (Maja Sticker Nordbrandt), Sponsor (Jessica Carlsson) and CTP (represented by its leader, Morten Ekstrøm).

**9.2 Collaboration partners**

The GCP unit at Copenhagen University Hospital, Bispebjerg Hospital is responsible for the monitoring.

The Department of Biostatistics - University of Copenhagen helps with statistics and randomisation.

Professor Derrick Silove‬

Psychiatry Research & Teaching Unit,

Liverpool Hospital,

University of New South Wales

Executive director Jorge Aroche,

The New South Wales Service for the treatment and rehabilitation of torture and trauma survivors (STARTTS).

CTP is part of a recently established international research network. STARTTS (New South Wales Service for the Treatment and Rehabilitation of Torture and Trauma Survivors) and Professor Silove are important parts of this network with many years of experience both in treatment and research within this field[[1]](#footnote-2). The cooperation opens new opportunities for the results of the present PhD study to be part of and influence the content of a future multicenter study which is due to start 3-4 years ahead. Moreover, this network provides an excellent opportunity for a longer period abroad as part of MN's PhD

**9.3 Time schedule**

| **Period** | **Project activity** |
| --- | --- |
| 1^st^ September 2013 – 1^st^ May 2015 | Collection and processing of patient data  Trial monitoring and correction  Data cleansing and statistical processing |
| 1^st^ May 2015 – 1^st^ September 2016 | Collection of data from the last patients  Completing data cleansing and statistical processing  Articles and PhD dissertation are written |

**9.4 Publications**

Positive as well as inconclusive or negative results will be published.

3 publications are planned corresponding to purpose 1, 2 and 4 of the study:

- The effect of physical activity as an add-on treatment to psychiatric treatment as usual for traumatised refugees.
- The psychological benefits of improvement of parameters of fitness when treating traumatised refugees with PTSD.
- The psychological benefits of a focus on body awareness when treating traumatised refugees with physical activity.

If the results cannot be published in a journal, they will be published at [www.clinicaltrials.gov](http://www.clinicaltrials.gov/) or [www.clinicaltrialsregister.eu](http://www.clinicaltrialsregister.eu/).

**9.5 Financing**

The investigator has been fully paid from CTP in the preparatory phase of the project.

The investigator is employed in the Mental Health Services in the Capital Region of Denmark as a clinical assistant. Aside from that, the investigator has no association with the contributors of the project.

The investigator will be registered as a PhD student at a relevant university starting from summer 2013 (The Faculty Research Committee found the project application qualified for enrolment at the Graduate School at Copenhagen University. This assessment is available by request from the applicant).

Applications regarding financial funding in terms of a scholarship for the investigator have been sent to University of Copenhagen and Region Hovedstadens Psykiatri. Applications regarding funding of the project as a whole have been sent to TrygFonden and Lundbeck Fonden. Funding for a physiotherapist have been applied to Psychiatric Centre Ballerup, from where the project in May 2013 has been granted the amount of a 5 months salary for a full time working physiotherapist. This equals around 141.000 kr (depending on the seniority of the physiotherapist that we hire for the job). Moreover, Oak Foundation and Fysioterapipraksisfonden will be applied during June 2013. If funding is achieved, information about the specific amount will be given to VEK.

**9.6 Reports**

At the end of the trial, the results will be published in articles as described above and in a PhD dissertation at the relevant university.

**10. References**

1. Masmas TN, Møller E, Buhmann C, Bunch V, Jensen JH, Hansen TN, et al. [Health status and degree of traumatisation among newly arrived asylum seeker--secondary publication]. Ugeskrift for laeger [Internet]. 2010 Jan [cited 2011 Apr 9];172(2):120–4. Available from: http://www.ncbi.nlm.nih.gov/pubmed/20376916

2. Chung RC, Kagawa-singer M. PREDICTORS OF PSYCHOLOGICAL DISTRESS AMONG SOUTHEAST ASIAN REFUGEES. Social science & medicine (1982) [Internet]. 1993 Mar [cited 2013 Jan 29];36(5):631–9. Available from: http://www.ncbi.nlm.nih.gov/pubmed/8456333

3. Sabin M, Nackerud L, Kaiser R. Factors associated with poor mental health among Guatemalan refugees living in Mexico 20 years after civil conflict. American medical association. 2003;290(5):635–42.

4. Carlson EB, Rosser-Hogan R. Cross-cultural response to trauma: a study of traumatic experiences and posttraumatic symptoms in Cambodian refugees. Journal of traumatic stress [Internet]. 1994 Jan [cited 2013 Jan 29];7(1):43–58. Available from: http://www.ncbi.nlm.nih.gov/pubmed/8044442

5. Shrestha NM, Sharma B, Van Ommeren M, Regmi S, Makaju R, Komproe I, et al. Impact of torture on refugees displaced within the developing world: symptomatology among Bhutanese refugees in Nepal. JAMA : the journal of the American Medical Association [Internet]. 1998 Aug 5 [cited 2013 Jan 29];280(5):443–8. Available from: http://www.ncbi.nlm.nih.gov/pubmed/9701080

6. Olsen DR, Montgomery E, Bøjholm S, Foldspang A. Prevalence of pain in the head, back and feet in refugees previously exposed to torture: a ten-year follow-up study. Disability and rehabilitation [Internet]. 2007 Jan 30 [cited 2013 Jan 29];29(2):163–71. Available from: http://www.ncbi.nlm.nih.gov/pubmed/17364766

7. Lawrence S, De Silva M, Henley R. Sports and games for post-traumatic stress disorder (PTSD). Cochrane database of systematic reviews (Online) [Internet]. 2010 Jan [cited 2011 Apr 6];(1):CD007171. Available from: http://www.ncbi.nlm.nih.gov/pubmed/20091620

8. Hetrick SE, Purcell R, Garner B, Parslow R. Combined pharmacotherapy and psychological therapies for post traumatic stress disorder (PTSD). Cochrane database of systematic reviews (Online) [Internet]. 2010 Jan [cited 2013 Feb 25];(7):CD007316. Available from: http://www.ncbi.nlm.nih.gov/pubmed/20614457

9. Bisson J, Andrew M. Psychological treatment of post-traumatic stress disorder (PTSD). Cochrane database of systematic reviews (Online) [Internet]. 2007 Jan [cited 2013 Feb 14];(3):CD003388. Available from: http://www.ncbi.nlm.nih.gov/pubmed/17636720

10. Stein DJ, Ipser JC, Seedat S. Pharmacotherapy for post traumatic stress disorder (PTSD). Cochrane database of systematic reviews (Online) [Internet]. 2006 Jan [cited 2013 Feb 18];(1):CD002795. Available from: http://www.ncbi.nlm.nih.gov/pubmed/16437445

11. Liedl A, Müller J, Morina N, Karl A, Denke C, Knaevelsrud C. Physical Activity within a CBT Intervention Improves Coping with Pain in Traumatized Refugees : Results of a Randomized Controlled Design. Pain Medicine. 2011;12:234–45.

12. Telles S, Singh N, Joshi M, Balkrishna A. Post traumatic stress symptoms and heart rate variability in Bihar flood survivors following yoga: a randomized controlled study. BMC psychiatry [Internet]. BioMed Central; 2010 Jan [cited 2012 Aug 20];10:18. Available from: /pmc/articles/PMC2836997/?report=abstract

13. Lund M, Sørensen J, Christensen J, Ølholm AM. MTV om behandling og rehabilitering af PTSD–herunder traumatiserede flygtninge. Region Syddanmark [Internet]. Center for Kvalitet. 2008 p. 9–375. Available from: http://scholar.google.com/scholar?hl=en&btnG=Search&q=intitle:MTV+om+behandling+og+rehabilitering+af+PTSD?herunder+traumatiserede+flygtninge.+Region+Syddanmark#0

14. National Institute of Clinical Exellence. Post-traumatic stress disorder (PTSD) The management of PTSD in adults and children in primary and secondary care [Internet]. Clinical Guideline 26, Developed by the National Collaborating Centre for Mental Health. National Institute for Clinical Excellence; 2005. Available from: http://www.ncbi.nlm.nih.gov/books/NBK56506/

15. Fysioterapi - www.rctfyn.dk. Fyns Amt; 2004 Jun 30 [cited 2011 May 5]; Available from: http://www.rctfyn.dk/wm150315

16. Behandling - www.cett.dk [Internet]. 2012 [cited 2013 Feb 15]. Available from: http://www.cett.dk/wm212122#behandling

17. Klinik for traumatiserede flygtninge, Region Midtjylland [Internet]. Psykiatri, Klinik for PTSD og Transkulturel. 2013 [cited 2011 May 5]. Available from: http://www.kftf.dk/index.php?option=com_content&task=view&id=19&Itemid=58

18. Den interdiciplinære tilgang [Internet]. Dignity, Dansk Institut Mod Tortur. 2013 [cited 2013 Feb 15]. Available from: http://www.dignityinstitute.dk/rehabilitering/den-interdiciplinaere-tilgang.aspx

19. Gyllensten AL, Hansson L, Ekdahl C. Outcome of Basic Body Awareness Therapy. A Randomized Controlled Study of Patients in Psychiatric Outpatient Care. Advances in Physiotherapy [Internet]. Informa UK Ltd  UK; 2003 Jan [cited 2011 Apr 9];5(4):179–90. Available from: http://informahealthcare.com/doi/abs/10.1080/14038109310012061

20. Cabral P, Meyer HB, Ames D. Effectiveness of yoga therapy as a complementary treatment for major psychiatric disorders: a meta-analysis. The primary care companion to CNS disorders [Internet]. Physicians Postgraduate Press, Inc.; 2011 Jan [cited 2012 Mar 10];13(4). Available from: /pmc/articles/PMC3219516/?report=abstract

21. Brown RP, Gerbarg PL. Sudarshan Kriya yogic breathing in the treatment of stress, anxiety, and depression: part I-neurophysiologic model. Journal of alternative and complementary medicine (New York, N.Y.) [Internet]. 2005 Feb [cited 2011 Apr 14];11(1):189–201. Available from: http://www.ncbi.nlm.nih.gov/pubmed/15750381

22. Hedlund L, Gyllensten AL. The experiences of basic body awareness therapy in patients with schizophrenia. Journal of bodywork and movement therapies [Internet]. 2010 Jul [cited 2013 Jan 22];14(3):245–54. Available from: http://www.ncbi.nlm.nih.gov/pubmed/20538222

23. De Moor MHM, Boomsma DI, Stubbe JH, Willemsen G, De Geus EJC. Testing causality in the association between regular exercise and symptoms of anxiety and depression. Archives of general psychiatry [Internet]. 2008 Aug;65(8):897–905. Available from: http://www.ncbi.nlm.nih.gov/pubmed/18678794

24. Friis S, Skatteboe UB, Hope MK, Vaglum P. Body awareness group therapy for patients with personality disorders. 2. Evaluation of the Body Awareness Rating Scale. Psychotherapy and psychosomatics [Internet]. 1989 Jan [cited 2013 Jan 21];51(1):18–24. Available from: http://www.ncbi.nlm.nih.gov/pubmed/2602528

25. Bräuninger I. The efficacy of dance movement therapy group on improvement of quality of life: A randomized controlled trial. The Arts in Psychotherapy [Internet]. Elsevier Ltd; 2012 Sep [cited 2013 Jan 30];39(4):296–303. Available from: http://linkinghub.elsevier.com/retrieve/pii/S0197455612000329

26. Porges SW. The polyvagal theory: phylogenetic substrates of a social nervous system. International journal of psychophysiology : official journal of the International Organization of Psychophysiology [Internet]. 2001 Oct;42(2):123–46. Available from: http://www.ncbi.nlm.nih.gov/pubmed/11587772

27. Craft LL, Perna FM. The Benefits of Exercise for the Clinically Depressed. Primary care companion to the Journal of clinical psychiatry [Internet]. 2004 Jan [cited 2010 Dec 6];6(3):104–11. Available from: http://www.pubmedcentral.nih.gov/articlerender.fcgi?artid=474733&tool=pmcentrez&rendertype=abstract

28. Dunn AL, Trivedi MH, Kampert JB, Clark CG, Chambliss HO. Exercise treatment for depression: efficacy and dose response. American journal of preventive medicine [Internet]. 2005 Jan [cited 2010 Aug 5];28(1):1–8. Available from: http://www.ncbi.nlm.nih.gov/pubmed/15626549

29. Mead G, Morley W, Campbell P, Carolyn A G, McMurdo M, Lawlor DA. Exercise for depression. Cochrane database of systematic reviews (Online) [Internet]. 2008 [cited 2011 Apr 6];(4):CD004366. Available from: http://www.ncbi.nlm.nih.gov/pubmed/18843656

30. Krogh J, Saltin B, Gluud C, Nordentoft M. The DEMO trial: a randomized, parallel-group, observer-blinded clinical trial of strength versus aerobic versus relaxation training for patients with mild to moderate depression. The Journal of clinical psychiatry. 2009 Jun;70(6):790–800.

31. Krogh J, Nordentoft M, Sterne J a C, Lawlor D a. The effect of exercise in clinically depressed adults: systematic review and meta-analysis of randomized controlled trials. The Journal of clinical psychiatry. 2011 Apr;72(4):529–38.

32. Manger TA, Motta RW. The impact of an exercise program on posttraumatic stress disorder, anxiety, and depression. International journal of emergency mental health [Internet]. 2005 Jan [cited 2011 Apr 4];7(1):49–57. Available from: http://www.ncbi.nlm.nih.gov/pubmed/15869081

33. King SJ, Wessel J, Bhambhani Y, Sholter D, Maksymowych W. The effects of exercise and education, individually or combined, in women with fibromyalgia. The Journal of rheumatology [Internet]. 2002 Dec [cited 2011 Apr 15];29(12):2620–7. Available from: http://www.ncbi.nlm.nih.gov/pubmed/12465163

34. Richards SCM, Scott DL. Prescribed exercise in people with fibromyalgia: parallel group randomised controlled trial. BMJ (Clinical research ed.) [Internet]. 2002 Jul 27 [cited 2011 Apr 15];325(7357):185. Available from: http://www.pubmedcentral.nih.gov/articlerender.fcgi?artid=117444&tool=pmcentrez&rendertype=abstract

35. Carson JW, Carson KM, Jones KD, Bennett RM, Wright CL, Mist SD. A pilot randomized controlled trial of the Yoga of Awareness program in the management of fibromyalgia. Pain [Internet]. 2010 Nov [cited 2011 Jan 27];151(2):530–9. Available from: http://www.ncbi.nlm.nih.gov/pubmed/20946990

36. Price CJ, McBride B, Hyerle L, Kivlahan DR. Mindful awareness in body-oriented therapy for female veterans with post-traumatic stress disorder taking prescription analgesics for chronic pain: a feasibility study. Alternative therapies in health and medicine [Internet]. NIH Public Access; 2007 [cited 2012 Sep 21];13(6):32–40. Available from: /pmc/articles/PMC3037268/?report=abstract

37. Malmgren-Olsson E, Armelius B, Armelius K. A Comparative outcome study of body awareness therapy, feldenkrais, and conventional physiotherapy for patients with nonspecific musculoskeletal disorders: changes in phychological symptoms, pain and self-image. Physiotherapy Theory and Practice [Internet]. 2001 [cited 2013 Jan 22];17(2):77–95. Available from: http://informahealthcare.com.ep.fjernadgang.kb.dk/toc/ptp/17/2

38. Malmgren-Olsson E-B, Bränholm I-B. A comparison between three physiotherapy approaches with regard to health-related factors in patients with non-specific musculoskeletal disorders. Disability and rehabilitation [Internet]. 2002 Apr 15 [cited 2011 May 5];24(6):308–17. Available from: http://www.ncbi.nlm.nih.gov/pubmed/12017464

39. Gard G. Body awareness therapy for patients with fibromyalgia and chronic pain. Disability and rehabilitation [Internet]. 2005 Jun 17 [cited 2011 May 5];27(12):725–8. Available from: http://www.ncbi.nlm.nih.gov/pubmed/16012065

40. Williams KA, Petronis J, Smith D, Goodrich D, Wu J, Ravi N, et al. Effect of Iyengar yoga therapy for chronic low back pain. Pain [Internet]. 2005 May [cited 2013 Feb 2];115(1-2):107–17. Available from: http://www.ncbi.nlm.nih.gov/pubmed/15836974

41. Trial AR, Garfinkel MS, Singhal A, Katz WA, Allan DA, Reshetar R, et al. Yoga-Based Intervention for Carpal Tunnel Syndrome. American Medical Association. 1998;280(18).

42. Sharp TJ, Harvey a G. Chronic pain and posttraumatic stress disorder: mutual maintenance? Clinical psychology review [Internet]. 2001 Aug [cited 2013 Feb 2];21(6):857–77. Available from: http://www.ncbi.nlm.nih.gov/pubmed/11497210

43. Jenewein J. Wittmann L. Moergeli H. Creutzig J. Schnyder U. Mutual influence of posttraumatic stress disorder symptoms and chronic pain among injured accident survivors: a longitudinal study. Journal of traumatic stress. 2009;540–8.

44. Liedl a, O’Donnell M, Creamer M, Silove D, McFarlane a, Knaevelsrud C, et al. Support for the mutual maintenance of pain and post-traumatic stress disorder symptoms. Psychological medicine [Internet]. 2010 Jul [cited 2013 Feb 3];40(7):1215–23. Available from: http://www.ncbi.nlm.nih.gov/pubmed/19811699

45. Meeusen R, Thorré K, Chaouloff F, Sarre S, De Meirleir K, Ebinger G, et al. Effects of tryptophan and/or acute running on extracellular 5-HT and 5-HIAA levels in the hippocampus of food-deprived rats. Brain research [Internet]. 1996 Nov 18 [cited 2011 Apr 16];740(1-2):245–52. Available from: http://www.ncbi.nlm.nih.gov/pubmed/8973821

46. Neeper SA, Gómez-Pinilla F, Choi J, Cotman CW. Physical activity increases mRNA for brain-derived neurotrophic factor and nerve growth factor in rat brain. Brain research [Internet]. 1996 Jul 8 [cited 2011 Apr 16];726(1-2):49–56. Available from: http://www.ncbi.nlm.nih.gov/pubmed/8836544

47. Angelopoulos TJ. Beta-endorphin immunoreactivity during high-intensity exercise with and without opiate blockade. European journal of applied physiology [Internet]. 2001 Nov [cited 2011 Apr 16];86(1):92–6. Available from: http://www.ncbi.nlm.nih.gov/pubmed/11820329

48. Hill EE, Zack E, Battaglini C, Viru M, Viru A, Hackney AC. Exercise and circulating cortisol levels: the intensity threshold effect. Journal of endocrinological investigation [Internet]. 2008 Jul [cited 2011 Apr 16];31(7):587–91. Available from: http://www.ncbi.nlm.nih.gov/pubmed/18787373

49. Harvey SB, Hotopf M, Overland S, Mykletun A. Physical activity and common mental disorders. The British journal of psychiatry : the journal of mental science [Internet]. 2010 Nov [cited 2010 Nov 3];197:357–64. Available from: http://www.ncbi.nlm.nih.gov/pubmed/21037212

50. Seals DR, Chase PB. Influence of physical training on heart rate variability and baroreflex circulatory control. Journal of applied physiology (Bethesda, Md. : 1985) [Internet]. 1989 Apr [cited 2011 Apr 17];66(4):1886–95. Available from: http://www.ncbi.nlm.nih.gov/pubmed/2732182

51. Rechlin T, Weis M, Spitzer A, Kaschka WP. Are affective disorders associated with alterations of heart rate variability? Journal of affective disorders [Internet]. 1994 Dec [cited 2011 Apr 19];32(4):271–5. Available from: http://www.ncbi.nlm.nih.gov/pubmed/7897091

52. Van der Kolk BA. Clinical implications of neuroscience research in PTSD. Annals of the New York Academy of Sciences [Internet]. 2006 Jul [cited 2010 Aug 1];1071:277–93. Available from: http://www.ncbi.nlm.nih.gov/pubmed/16891578

53. Dekker JM, Schouten EG, Klootwijk P, Pool J, Swenne CA, Kromhout D. Heart rate variability from short electrocardiographic recordings predicts mortality from all causes in middle-aged and elderly men. The Zutphen Study. American journal of epidemiology [Internet]. 1997 May 15 [cited 2011 Apr 19];145(10):899–908. Available from: http://www.ncbi.nlm.nih.gov/pubmed/9149661

54. McCraty R, Atkinson M, Tiller WA, Rein G, Watkins AD. The effects of emotions on short-term power spectrum analysis of heart rate variability . The American journal of cardiology [Internet]. 1995 Nov 15 [cited 2011 Apr 19];76(14):1089–93. Available from: http://www.ncbi.nlm.nih.gov/pubmed/7484873

55. Porges SW, Doussard-Roosevelt JA, Portales AL, Greenspan SI. Infant regulation of the vagal “brake” predicts child behavior problems: a psychobiological model of social behavior. Developmental psychobiology [Internet]. 1996 Dec [cited 2011 Apr 19];29(8):697–712. Available from: http://www.ncbi.nlm.nih.gov/pubmed/8958482

56. Cohen JA, Perel JM, DeBellis M, Friedman M, Putnam F. Treating traumatized children: clinical implications of the psychobiology of posttraumatic stress disorder. Trauma, Violence & Abuse [Internet]. 2002 [cited 2011 Apr 19];3(2):91–108. Available from: http://www.nctsnet.org/nctsn_assets/Articles/28.pdf

57. De Bellis MD, Baum AS, Birmaher B, Ryan ND. Urinary catecholamine excretion in childhood overanxious and posttraumatic stress disorders. Annals of the New York Academy of Sciences [Internet]. 1997 Jun 21 [cited 2013 Feb 15];821:451–5. Available from: http://www.ncbi.nlm.nih.gov/pubmed/9238227

58. De Bellis MD, Keshavan MS, Clark DB, Casey BJ, Giedd JN, Boring AM, et al. A.E. Bennett Research Award. Developmental traumatology. Part II: Brain development. Biological psychiatry [Internet]. 1999 May 15 [cited 2013 Feb 16];45(10):1271–84. Available from: http://www.ncbi.nlm.nih.gov/pubmed/10349033

59. Pitman RK. Post-traumatic stress disorder, hormones, and memory. Biological psychiatry [Internet]. 1989 Jul [cited 2013 Feb 16];26(3):221–3. Available from: http://www.ncbi.nlm.nih.gov/pubmed/2545287

60. Buckley TC, Holohan D, Greif JL, Bedard M, Suvak M. Twenty-four-hour ambulatory assessment of heart rate and blood pressure in chronic PTSD and non-PTSD veterans. Journal of traumatic stress [Internet]. 2004 Apr [cited 2011 Apr 20];17(2):163–71. Available from: http://www.ncbi.nlm.nih.gov/pubmed/15141790

61. Muraoka MY, Carlson JG, Chemtob CM. Twenty-four-hour ambulatory blood pressure and heart rate monitoring in combat-related posttraumatic stress disorder. Journal of traumatic stress [Internet]. Springer Netherlands; 1998 Jul [cited 2011 Apr 20];11(3):473–84. Available from: http://www.springerlink.com/content/h68w7755nn347764/

62. Woodward SH, Arsenault NJ, Voelker K, Nguyen T, Lynch J, Skultety K, et al. Autonomic Activation During Sleep in Posttraumatic Stress Disorder and Panic: A Mattress Actigraphic Study. Biological psychiatry [Internet]. Elsevier; 2009 [cited 2011 Apr 4];66(1):41–6. Available from: http://linkinghub.elsevier.com/retrieve/pii/S0006322309000304

63. Berntson GG, Bigger JT, Eckberg DL, Grossman P, Kaufmann PG, Malik M, et al. Heart rate variability: origins, methods, and interpretive caveats. Psychophysiology [Internet]. 1997 Nov [cited 2013 Feb 14];34(6):623–48. Available from: http://www.ncbi.nlm.nih.gov/pubmed/9401419

64. Hopper JW, Spinazzola J, Simpson WB, Van der Kolk B a. Preliminary evidence of parasympathetic influence on basal heart rate in posttraumatic stress disorder. Journal of psychosomatic research [Internet]. 2006 Jan [cited 2011 Apr 6];60(1):83–90. Available from: http://www.sciencedirect.com.ep.fjernadgang.kb.dk/science?_ob=ArticleURL&_udi=B6T8V-4HW8YTT-H&_user=9843631&_coverDate=01/31/2006&_rdoc=1&_fmt=high&_orig=gateway&_origin=gateway&_sort=d&_docanchor=&view=c&_acct=C000034378&_version=1&_urlVersion=0&_userid=9843631&md5=02a6d13a56c6ea784bb76b5ee26fca5e&searchtype=a

65. Devinsky O, Morrell MJ, Vogt BA. Contributions of anterior cingulate cortex to behaviour. Brain : a journal of neurology [Internet]. 1995 Feb [cited 2013 Feb 25];118 ( Pt 1:279–306. Available from: http://www.ncbi.nlm.nih.gov/pubmed/7895011

66. Lanius RA, Bluhm R, Lanius U, Pain C. A review of neuroimaging studies in PTSD: heterogeneity of response to symptom provocation. Journal of psychiatric research [Internet]. 2006 Dec [cited 2013 Feb 1];40(8):709–29. Available from: http://www.ncbi.nlm.nih.gov/pubmed/16214172

67. Morgan MA, Romanski LM, LeDoux JE. Extinction of emotional learning: contribution of medial prefrontal cortex. Neuroscience letters [Internet]. 1993 Nov 26 [cited 2013 Feb 25];163(1):109–13. Available from: http://www.ncbi.nlm.nih.gov/pubmed/8295722

68. Shin LM, Whalen PJ, Pitman RK, Bush G, Macklin ML, Lasko NB, et al. An fMRI study of anterior cingulate function in posttraumatic stress disorder. Biological psychiatry [Internet]. 2001 Dec 15 [cited 2013 Feb 25];50(12):932–42. Available from: http://www.ncbi.nlm.nih.gov/pubmed/11750889

69. Markowitsch HJ, Kessler J, Weber-Luxenburger G, Van der Ven C, Albers M, Heiss WD. Neuroimaging and behavioral correlates of recovery from mnestic block syndrome and other cognitive deteriorations. Neuropsychiatry, neuropsychology, and behavioral neurology [Internet]. 2000 Jan [cited 2013 Feb 25];13(1):60–6. Available from: http://www.ncbi.nlm.nih.gov/pubmed/10645738

70. Vasterling JJ, Brailey K, Constans JI, Sutker PB. Attention and Memory Dysfunction in Posttraumatic Stress Disorder. Neuropsychology. 1998;12(1):125–33.

71. Roxendal G. Body awareness therapy and the body awareness scale : treatment and evaluation in psychiatric physiotherapy [Internet]. Sweden. 1985 [cited 2013 Jan 21]. p. 2–60. Available from: http://www.ibk.nu/abstracts/avhandling_roxendal.pdf

72. Gyllensten A. Basic Body Awareness Therapy: Assessment, Treatment and Interaction [Internet]. Lund: Studentliteratur. Lund University; 2001 [cited 2013 May 8]. Available from: http://en.scientificcommons.org/7602613

73. Grahn B, Ekdahl C, Borgquist L. Effects of a multidisciplinary rehabilitation programme on health-related quality of life in patients with prolonged musculoskeletal disorders; a 6-month follow-up of a prospective controlled study. Disability and Rehabilitation [Internet]. 1998 Aug [cited 2013 Jan 25];20(8):285–97. Available from: http://www.ncbi.nlm.nih.gov/pubmed/9651687

74. Price CJ, Thompson EA. Measuring Dimensions of Body Connection: Body Awareness and Bodily Dissociation. Journal Of Alternative And Complementary Medicine. 2007;13(9):945–53.

75. Margoles M, Weiner R. Chronic Pain: Assessment, Diagnosis and Management. Danvers, MA: CRC Press; 1999.

76. Taylor GJ. Affects, trauma, and mechanisms of symptom formation: a tribute to John C. Nemiah, MD (1918-2009). Psychotherapy and psychosomatics [Internet]. 2010 Jan [cited 2013 Apr 25];79(6):339–49. Available from: http://www.ncbi.nlm.nih.gov/pubmed/20733344

77. Bob P. Pain, dissociation and subliminal self-representations. Consciousness and cognition [Internet]. 2008 Mar [cited 2013 Mar 18];17(1):355–69. Available from: http://www.ncbi.nlm.nih.gov/pubmed/18207424

78. Schulz KF, Altman DG, Moher D, Group C. Annals of Internal Medicine Academia and Clinic CONSORT 2010 Statement : Updated Guidelines for Reporting Parallel Group Randomized Trials TO CONSORT. Annals of Internal Medicine. 2010;152(11):1–8.

79. Mollica RF, Caspi-Yavin Y, Bollini P, Truong T, Tor S, Lavelle J. The Harvard Trauma Questionnaire. Validating a cross-cultural instrument for measuring torture, trauma, and posttraumatic stress disorder in Indochinese refugees. The Journal of nervous and mental disease [Internet]. 1992 Feb [cited 2013 Apr 28];180(2):111–6. Available from: http://www.ncbi.nlm.nih.gov/pubmed/1737972

80. Mollica RF, Wyshak G, De Marneffe D, Khuon F, Lavelle J. Indochinese versions of the Hopkins Symptom Checklist-25: a screening instrument for the psychiatric care of refugees. The American journal of psychiatry [Internet]. 1987 Apr [cited 2013 Feb 3];144(4):497–500. Available from: http://www.ncbi.nlm.nih.gov/pubmed/3565621

81. HAMILTON M. A rating scale for depression. Journal of neurology, neurosurgery, and psychiatry [Internet]. 1960 Mar [cited 2013 Jan 31];23:56–62. Available from: http://www.pubmedcentral.nih.gov/articlerender.fcgi?artid=495331&tool=pmcentrez&rendertype=abstract

82. Blom EH, Bech P, Högberg G, Larsson JO, Serlachius E. Screening for depressed mood in an adolescent psychiatric context by brief self-assessment scales -- testing psychometric validity of WHO-5 and BDI-6 indices by latent trait analyses. Health and quality of life outcomes [Internet]. 2012 Dec 11 [cited 2013 Feb 3];10(1):149. Available from: http://www.ncbi.nlm.nih.gov/pubmed/23227908

83. Grootenboer EM V, Giltay EJ, Van der Lem R, Van Veen T, Van der Wee NJA, Zitman FG. Reliability and validity of the Global Assessment of Functioning Scale in clinical outpatients with depressive disorders. Journal of evaluation in clinical practice [Internet]. 2012 Apr [cited 2013 Feb 27];18(2):502–7. Available from: http://www.ncbi.nlm.nih.gov/pubmed/21223457

84. Forbes DA. Goal Attainment Scaling. A responsive measure of client outcomes. Journal of gerontological nursing [Internet]. 1998 Dec [cited 2013 Feb 27];24(12):34–40. Available from: http://www.ncbi.nlm.nih.gov/pubmed/10025309

85. Arbuckle R, Frye MA, Brecher M, Paulsson B, Rajagopalan K, Palmer S, et al. The psychometric validation of the Sheehan Disability Scale (SDS) in patients with bipolar disorder. Psychiatry research [Internet]. 2009 Jan 30 [cited 2013 Feb 27];165(1-2):163–74. Available from: http://www.ncbi.nlm.nih.gov/pubmed/19042030

86. Elklit A, Pedersen SS, Jind L. The Crises Support Scale: psychometric qualities and further validation. Personality and Individual Differences [Internet]. 2001 [cited 2013 May 8];31:1291–302. Available from: http://static.sdu.dk/mediafiles/8/E/0/%7B8E0F0B02-9707-41E9-B973-F5A9E4BC5D93%7D2001_Artikel_4.pdf

87. Boonstra AM, Schiphorst Preuper HR, Reneman MF, Posthumus JB, Stewart RE. Reliability and validity of the visual analogue scale for disability in patients with chronic musculoskeletal pain. International journal of rehabilitation research. Internationale Zeitschrift für Rehabilitationsforschung. Revue internationale de recherches de réadaptation [Internet]. 2008 Jun [cited 2013 Feb 27];31(2):165–9. Available from: http://www.ncbi.nlm.nih.gov/pubmed/18467932

88. Cleeland CS, Ryan KM. Pain assessment: global use of the Brief Pain Inventory. Annals of the Academy of Medicine, Singapore [Internet]. 1994 Mar [cited 2013 Feb 4];23(2):129–38. Available from: http://www.ncbi.nlm.nih.gov/pubmed/8080219

89. Atkinson TM, Rosenfeld BD, Sit L, Mendoza TR, Fruscione M, Lavene D, et al. Using confirmatory factor analysis to evaluate construct validity of the Brief Pain Inventory (BPI). Journal of pain and symptom management [Internet]. 2011 Mar [cited 2013 Feb 3];41(3):558–65. Available from: http://www.pubmedcentral.nih.gov/articlerender.fcgi?artid=3062715&tool=pmcentrez&rendertype=abstract

90. Mehling WE, Price C, Daubenmier JJ, Acree M, Bartmess E, Stewart A. The Multidimensional Assessment of Interoceptive Awareness (MAIA). PloS one [Internet]. 2012 Jan [cited 2013 Feb 8];7(11):e48230. Available from: http://www.pubmedcentral.nih.gov/articlerender.fcgi?artid=3486814&tool=pmcentrez&rendertype=abstract

91. Marchetti GF, Whitney SL. Construction and validation of the 4-item dynamic gait index. Physical therapy [Internet]. 2006 Dec [cited 2013 Feb 28];86(12):1651–60. Available from: http://www.ncbi.nlm.nih.gov/pubmed/17062644

92. Hansen H. Senior Fitness Test - Danske Fysioterapeuter [Internet]. 2013 [cited 2013 Feb 28]. Available from: http://fysio.dk/fafo/Maleredskaber/Maleredskaber-alfabetisk/Senior-Fitness-Test/

93. Hansen H. Dynamic Gait Index (Dynamisk Gangindeks) * - Danske Fysioterapeuter [Internet]. 2012 [cited 2013 Feb 28]. Available from: http://fysio.dk/fafo/Maleredskaber/Maleredskaber-alfabetisk/Dynamic-Gait-Index-Dynamisk-Gangindeks/

94. Macri EM, Lewis JA, Khan KM, Ashe MC, De Morton NA. The de morton mobility index: normative data for a clinically useful mobility instrument. Journal of aging research [Internet]. 2012 Jan [cited 2013 Feb 28];2012:353252. Available from: http://www.pubmedcentral.nih.gov/articlerender.fcgi?artid=3440954&tool=pmcentrez&rendertype=abstract

95. Hollifield M, Warner TD, Lian N, Krakow B, Jenkins JH, Kesler J, et al. Measuring trauma and health status in refugees: a critical review. JAMA : the journal of the American Medical Association [Internet]. 2002 Aug 7 [cited 2013 Feb 3];288(5):611–21. Available from: http://www.ncbi.nlm.nih.gov/pubmed/12150673

1. Letter of recommendation from Professor Silove is available by request from the applicant. [↑](#footnote-ref-2)
