# Supplementary material for: Trauma-affected refugees treated with basic body awareness therapy or mixed physical activity as augmentation to treatment as usual—A pragmatic randomised controlled trial
Source: PLoS One. 2020 Mar 12;15(3):e0230300. doi: 10.1371/journal.pone.0230300 (PMC7067472; doi:10.1371/journal.pone.0230300)
Supplement: Attachment — Submitted filename: review denen.docx [file pone.0230300.s005.docx]

Thus, the current meta-analysis builds considerably on the previous Cochrane review of sports and games for PTSD (Lawrenceetal.,2010) which did not identify any RCTs at the time examining exercise interventions for PTSD. Of the four included trials, two used a yoga-based intervention(Kolk et al.,2014; Mitchell et al.,2014) which were eligible for inclusion under the definition of physical activity as any bodily movement produced by skeletal muscle requiring energy expenditure (Caspersen et al.,1985). Given that yoga cannot be classified as either strictly a cardiorespiratory or resistance based activity,the results of this review demonstrate that both types of activity are likely beneficial.

________________________________________________________________________________

First, I would like to compliment the authors with their study, which has been conducted thoroughly. The subject of the study is extremely relevant since it evaluates treatment for a target group that suffers tremendously and does not receive a lot of attention. It is of the utmost importance that the authors state that results from other groups suffering from PTSD are not always applicable to refugees with trauma-related disorders.

Furthermore, the addition of body- and movement-related interventions is of importance when dealing with trauma-related disorders, in which the body as locus of control is so severely damaged.

The manuscript is highly readable.

My questions and remarks are the following:

**Abstract**

Line 22: It would be better to define the aim somewhat broader, thereby differentiating the two forms of physical activity and making clear already that there are 3 arms.

Line 28/29: these lines should be rewritten, making clear that both add-on treatments are individual physiotherapy treatments.

Conclusions: could some lines as to the why of the lack of difference be added? (discussion).

It think this would better reflect the importance of the study, although results do not show differences to TAU.

**Introduction:**

I would suggest to skip the reference to the retracted article (lines 60-65).

I would suggest to make a difference between BBAT and mixed physical activity.

The text now mentions BBAT as a body-awareness oriented therapy in line 67, but in the lines following the effect of physical activity is the main issue.

Then the aim of the study is stated as evaluating if either BBAT or mixed physical activity would increase the treatment effect. But I would think there are thoughts about the differences of the effect and it would strengthen the article to articulate some hypotheses regarding the effect of targeting body awareness in trauma. See also e.g. Scheffers et al., 2017, Price et al. NOG OPZOEKEN

**Participants**

It is not clear to me whether the patients are all outpatients, or may be in day-treatment or in-patients

Line 117: patients who did not wish to participate in this study were offered TAU. Does this mean they were not part of the 110 participants in TAU?? Otherwise it should be made clear in the following section about randomization that these patients (and how many) take part.

**Interventions:**

These remarks are connected to my other remarks about the difference between BBAT, especially its specific therapeutic goals, and mixed physical activity.

Therefore, I would recommend to describe the two arms separately and not under the common denominator of physical activity. By this subdivision the content of the manuscript will be more according to what is suggested in the title of the manuscript.

**Results**

Lines 244/245

It seems rather obvious that, given a total time of ten months and an average attendance of ten sessions, so physiotherapy once a month, no effect is to be expected. I think this point should get more attention in the discussion. A heightened frequency and more focus on attendance (attendance being a general problem, see also the literature on physical activity for people with schizophrenia for example) could be a recommendation for the future.

How many sessions were there for the intervention groups? I read 20 weekly sessions starting in phase one (line 159). Does this mean a total of 20 sessions?

Line 250: was the percentage of comorbid depression established by the medical doctor according to ICD 10 criteria compared to the Hamilton depression scores? This seems sensible since the comorbid depression is surprisingly high (even given the fact that depression is the main comorbid disorder in PTSD).

Table 2: The fitness results and the MAIA results are absent in this table, while they are crucial for the measurement of the add-on effects. So, it is not clear which measures are for what reason in the Supporting information.

**Discussion**

Lines 315-316: I would suggest to delete the remark about the interventions in this study not being different from those in the Rosenbaum review. Interventions as well as populations are all very different in the Rosenbaum review, and are also different from the ones in the present study.

345: duration is long, but frequency is a weakness!

348: frequency of once a week would suggest 10 months = possibly 40 sessions!

356-358 I would suggest to skip these lines and possibly look for other references. The authors rely too much on Rosenbaum et al.

Lines 368-371: the add-ons are different because they use body- and movement interventions and it is strange to suggest that the bodily aspects were already met in the TAU.

Lines 374 and further: I have the impression the authors are disappointed, which I can imagine, but I think it would be worthwhile to think about other options, such as frequency of the add-on treatment, motivational issues, etc.

**Impact on future treatment**.

The first lines should be deleted. Adding physical activity 10 times in a ten month treatment and having no effect, does not justify this conclusion.

**General comments**

Given the fact that two of the intervention studied pertain physical activity, it seems important to define physical activity (see also Rosenbaum et al.). Of the four included trials in the Rosenbaum review, two used a yoga-based intervention.

It is unclear why two different physical activity interventions are offered. It is stated that it is important to evaluate differences between the two, but why are two different activities offered in the first place?

Rosenbaum et al. define physical activity as any bodily movement produced by skeletal muscle requiring energy expenditure, thereby citing Caspersen et al. (1985).

However, BBAT could also be regarded as a body- and movement psychotherapeutic intervention, not aiming in the first place at a better physical activity but on ameliorating body-awareness as first aim. See e.g. Erikson et al. (2007) : Body awareness therapy represents a body-oriented physiotherapeutic approach focusing on both the physical and psychological aspects of body dysfunctions; see also the description in Catalan-Matamoros et al. (2010) on BBAT.

In the title of the manuscript it says: basic body awareness therapy or mixed physical activity, giving the impression that these are differently aimed interventions. However, further in the manuscript they are both shared under physical activity.
