# Supplementary material for: Trauma-affected refugees treated with basic body awareness therapy or mixed physical activity as augmentation to treatment as usual—A pragmatic randomised controlled trial
Source: PLoS One. 2020 Mar 12;15(3):e0230300. doi: 10.1371/journal.pone.0230300 (PMC7067472; doi:10.1371/journal.pone.0230300)
Supplement: Attachment — Submitted filename: Response to Reviewers.docx [file pone.0230300.s007.docx]

PONE-D-19-16080
Trauma-affected refugees treated with basic body awareness therapy or mixed physical activity as augmentation to treatment as usual – a pragmatic randomised controlled trial
PLOS ONE

Dear Editor and reviewers

Thank you for offering the opportunity to further improve the manuscript based on your comments

Review Comments to the Author (after first revision)

Reviewer #1: As I said in the first review, the research findings did not support the idea of adding physical activity programme to TAU for refugees affected by PTSD. It appears not important to define the physical activity the authors are talking about.

*1. Response: Adding a definition of physical activity was a request by Reviewer#2 and therefore kept in the manuscript.*

Reviewer #2: The manuscript has been largely improved! It has been made clear why two different categories of physical activity have been chosen. Also, the differences between structured exercise and mind-body interventions have been outlined in more detail. Next, other issues I raised in my comments have been addressed satisfactorily. Below I state some remaining, mostly small, questions and comments.

2.1 General remark: The manuscript still contains several typos and double spaces. I addressed some below, but I advise to re-read the manuscript.

*2.1 Response: The manuscript has been carefully re-read to detect typos and double spaces.*

2.2 Abstract:
Lines 41-44
The authors now state: ‘A large number of the participants in the study have a chronic mental condition, often difficult to treat.’
However, they do not explain the link between the lack of effect related to PA and the chronic mental condition. So, this phrase does not add much in terms of explanations or hypotheses.

*2.2 Response: We do agree that as it stands this sentence does not clearly explain the link. However, due to a limitation in number of words in the abstract (word count 309 with a limit of 300) and as the question of chronicity is elaborated in the Discussion we have now chosen to delete the sentence in the abstract.*

2.3 Line 54
‘postmigration’ should be post-migration

*2.3 Response: This has been corrected.*

2.4 Line 65
‘andthe suggests’ should be corrected

*2.4 Response: “the” has been deleted.*

2.5 Line 67
mind body interventions should be mind-body interventions

*2.5 Response: This has been corrected.*

2.6 Lines 80-81
‘Physical activity in this study, was defined as suggested by Caspersen et al. “any bodily movement produced by skeletal muscles that results in energy expenditure ” [13]. ‘
I think after Caspersen et al. the word ‘as’ is missing. Furthermore, because this is a quotation, a page number should be added.

*2.6 Response: The word “as” has now been added. The page number has been added just after the in-text citation.*

2.7 ‘Further’ PA can be divided in etc. should be Furthermore

*2.7 Response: Further has been changed to Furthermore.*

2.8 Lines 151/152
‘Patients who did not wish to participate in this study were offered treatment corresponding to TAU’
I would say: ‘similar’ to TAU, but I am not a native speaker

*2.8 Response: The sentence has now been changed to: “Patients who did not wish to participate in this study were offered treatment* *as described in the TAU manual”.*

2.9 Line 211
(e.g.. body scan, rotation, the wave).
I suggest to delete these examples, because for the lay person, they do not provide an explanation and only rise more questions. May be apart from the body scan, but a body scan is not a typical BBAT exercise.

*2.9 Response: To avoid confusion we have now deleted “e.g.. body scan, rotation, the wave” as suggested.*

2.10 Line 216
everyday-like tool should be tools

*2.10 Response: Everyday-like tools has been changed to tools.*

2.11 Line 249
‘ i.e two data points’.
I think this information is reduntant.

*2.11 Response: “i.e two data points” was added as a response to a question regarding number of data points made by Reviewer #3 in the first review and is therefore kept in this revision.*

2.12 Line 366
‘primary and on many secondary outcomes’
Many secondary outcomes is rather vague.

*2.12 Response: We agree and “many” has now been changed to the specific number (four) and the names of the outcomes have been added.*

“…*on the primary and on four of the secondary outcomes (WHO, GAF-S, HoNOS, MAIA -not worrying)…”*

2.13 Line 370
4 studies should be four studies

*2.13 Response: This has been corrected to four studies.*

2.14 Lines 380-381
‘Several studies have previously suggested that treatment effects in
trauma-affected refugees are typically smaller than in other trauma-affected populations[24-26].’
These studies have been conducted by some of the present researchers. Therefore, if available, It would be good to add another reference, from another country/group of researchers.

*2.14 Response: As requested we have added a reference from a separate group of researchers (Ter Heide et al.,* *Eye movement desensitisation and reprocessing therapy v. stabilisation as usual for refugees: randomised controlled trial, Br J Psychiatry 2016).*

2.15 Lines 405-406
‘By using individual treatment sessions, no potential spillover effect of a group could blur the results.’
Did the authors also consider the possibility that a groupwise treatment could enhance the effects?
I would like to point out that the motivational intervention cited by the authors in line 436 (reference 46) is also a group intervention !

*2.15 Response: We did consider the possible advantages of group sessions. However, there was a main reason for not choosing group treatment which is now explained: “Additionally, BBAT as method was prior to this study tested in a pilot study on the same target group, and the method was found satisfactory and acceptable. In the pilot study, BBAT was offered in group sessions, which was the reason for several patients not wishing to participate in the study. In the present study we therefore avoided group sessions as this had been reported to be an obstacle for participation.”*

2.16 Line 408
lack for difference should be lack of difference

*2.16 Response: This has been corrected.*

Reviewer #4: The manuscript entitled 'Trauma-affected refugees treated with basic body awareness therapy or mixed physical activity as augmentation to treatment as usual – a pragmatic randomised controlled trial' with the aim to investigate if adding either BBAT or mixed physical activity to the treatment as usual (TAU) for trauma-affected refugees with PTSD would increase the treatment effect compared to TAU alone.

Comments

4.1 Questionnaires
The language version that was used by the patients to be cited and referenced for each questionnaire.

*4.1 Response: As stated in page 11: “All self-administered outcomes were available in the five main languages of the patients (Danish, Arabic, English, Bosnian/Serbo-Croatian and Farsi).”. There are only few references for the different language versions of the included questionnaires. These have now been included in the section Outcomes (Methods).*

4.2 For the baseline characteristics, the breakdown of education level to be provided. A statement on whether they are able to read/write and understand the questionnaire to be stated.

*4.2 Response: In the data material the education variable contains information on number of years of education in the home country (<2 years, 2-5 years, >5-10 years, >10-15 years and more than >15 years). This more detailed presentation of data on education has been inserted in table 1.*

*Most participants were able to read and fill in the questionnaires themselves. “**There were a few illiterate (number unknown) participants and also a few participants where the questionnaires had not been translated to their language. These participants were assisted by an interpreter.” This has been added in Outcomes (Methods), page 11. We have also added a sentence regarding assistance by interpreter during treatment at the end of the section Participants (Methods), page 8:* “*All patients in need of an interpreter received this assistance and if possible, the same interpreter was used throughout the treatment.”*

4.3 The results presentations should place more emphasis on the comparison of pre and post treatment of each group rather than focusing on the group difference at pre treatment and post treatment respectively altogether. Even though there was no significant different at baseline between the groups, the figures are still different from each other. This may affect on the post treatment value.

*4.3 Response: We find that the current table 2 presents all relevant means and pre-post treatment differences. Even though post-treatment group differences are often used as the primary outcome in randomised trials, pre-treatment group differences might affect the post-treatment group differences in absolute scores, and even though p-values for these differences are provided, the reviewer is right to stress the pre-post treatment differences in each group and in particular, the group differences in pre-post treatment changes. The latter group differences are likely to be little affected by pre-treatment differences and are an important test of different effects of the three treatment interventions.*

*As an alternative we have also analysed the data using a regression model with indicator variables for the three groups and the pre-treatment score as independent variable and the post-treatment score as outcome variable. This analysis – which fully adjusted for pre-treatment differences among the three groups – showed no significant group differences in post-treatment means, confirming the results of the mixed model analysis.*

4.4 Also based on CONSORT guidelines, all baseline comparison to be avoided.

*4.4 Response: We have now deleted the following sentence regarding baseline comparisons: “No significant group differences were seen at baseline on any of the characteristics listed in Table 1.” We have furthermore (for the same reason) deleted the sentence: 'There were no significant or borderline significant group differences among the data above” in table 1.*

4.5 Page 11 Line 227, for the sentence ' pre treatment interview, shortly before initiating phase two and at the last treatment' the word post treatment to be used.

*4.5 Response: The word post-treatment is now used as suggested.*

4.6 Page 11 Line 244-246, the outcome variable used for the sample size calculation to be stated. Other information to be provided e.g. alpha, mean, which two groups were used, software, formula etc. Post hoc multiple comparison method to be considered in the sample size calculation. There was no information on the attrition rates if it were taken into consideration of the sample size calculation.

*4.6 Response: The power calculations were conducted with regard to the HTQ, and this has been made more clear in the revised version of the paragraph where we also include the alpha level and a comment on multiple comparison – although due to lack of significant overall effects comparison of means was not used for the main outcomes. Concerning attrition rates the manuscript makes clear that power should be sufficient with about 200 participants, and that the study – because of expected attrition - was planned to include 250 participants, but that 338 patients were in fact included.*

*The revised paragraph on power now reads:*

*“Power calculations were conducted with regard to the primary outcome HTQ. The study was initially planned to include approximately 250 patients as a conservative estimate of 200 participants eligible for intention-to-treat analysis with about 65 in each of the three groups would provide power of 81% to detect a group difference corresponding to a standardised difference of ½ SD (Cohen’s d) and power to detect a difference of 1 SD of close to 100% (using a 5% level of significance and planning to use contrasts to compare group means in case of a significant overall test of group differences). A difference of less than ½ SD was considered less relevant from a clinical perspective. However, due to a smaller number than expected of male patients with a high HTQ score (which we stratified for in accordance with the average level of HTQ-score in previous RCTs conducted at CTP [24,25]) a larger number was included. Thus, the final number of included patients was 338.”*

4.7 Page 12 Line 250 - 251, the sentences to be placed after Line 264.

*4.7 Response: These two sentences have been moved to the end of “Statistical analysis” as suggested.*

4.8 Page 12 Line 253, 1 tailed or 2 tailed test to be stated. Fishers exact test to be rewritten as Fisher's exact test. The level of acceptance significance to be stated.

*4.8 Response: In the statistical paragraph the following sentence has been included in the revised version of the manuscript: Two-tailed tests with a 5% level of significance were used in all statistical tests. In addition, the spelling of Fisher’s exact test has been corrected*

4.9 Page 13 Table 1, at least one decimal point for percentages to be provided. The symbol % to be omitted since it has been indicated on the top. w.loss to be spelled out. F.62.0 to be denoted in the table footnote. The sentence 'There were no significant or borderline significant group differences among the data above' to be break up and denoted in table footnote. Symbol N for All, group C, B and M on to[ of table.

4.9 Response:

- One decimal is now provided for all the percentages
- The symbol % has been taken out
- “Cranial trauma with loss of consciousness” is now spelled out.
- The code F62.0 has been deleted from the table as the table does not contain other codes for diagnoses.
- The sentence on significant group differences is taken out (please see response 4.4)
- Symbol N is now inserted for All, group C, B and M in the top of the table.

4.10 Table 2, effect size could be explored. Symbol <= to be replaced with ≤. Statistical test to be denoted in the table footnote. Decimal points for p value to be standardized.

*4.10 Response:*

- *Effect size: Since table 2 does not show a single significant group difference in absolute post-treatment scores or in changes between pre- and post-treatment scores, it hardly makes sense to calculate effect size. However, several measures show significant changes from pre- to post-treatment scores. Since there are no significant group differences in these changes, it makes sense to calculate estimates of effect size based on the full sample. The range of these effect sizes vary from 0.07 (BPI interference) to 0.57 (GAF-S), using the baseline SD to standardise the changes from pre- to post-treatment. Corresponding to the levels of significance in the table, the largest estimated effect sizes were 0.48 SD (HTQ), 0.53 SD (WHO-5) and 0.57 SD (GAF-S). Since these changes are unrelated to the investigated interventions, they may reflect effects of TAU or non-treatment related factors. Because the focus of the paper is on the effects of the interventions, we have not found it appropriate to discuss these effect estimates in the revised paper, but this can be included if the editor finds that it should.*
- *The symbol <= has been replaced with ≤ in table 2, S1 and S2*
- *Denotation of statistical test: Manuscript table 2 includes the footnote below. We find that this is a very detailed explanation and see no need to expand this description. Should the editor disagree we are of course willing to do so.*

“All means and SEs in table 2 are mixed model estimates and so are the p-values. The p-values in the pre- and post-treatment columns refer to an overall test of the significance mean differences between the three groups (corresponding to an ANOVA F-test). The p-values in the column showing pre-post treatment differences refer to an overall test of the significance of group differences in mean pre-post treatment differences (this test of group differences in pre-post treatment difference corresponds to the statistical test of the time by intervention interaction). Finally, the column with p-values shows p-values corresponding to the pre-post treatment mean difference in each of the three groups.”

- *The decimal points for the p value have been standardised as requested.*

4.11 S1 Table & S2 Table, few figures with 1 decimal point (to be consistent with 2 decimal points).

*4.11 Response: S1 and S2 have now been carefully looked through and corrected as to inconsistency in number of decimals.*

4.12 References list did not conform to the journal format.

*4.12 Response: The reference list has been corrected to conform with the journal format.*
